# North Asian population relationships in a global context

**DOI:** 10.1038/s41598-022-10706-x

**Published:** 2022-05-04

**Authors:** Kenneth K. Kidd, Baigalmaa Evsanaa, Ariunaa Togtokh, Jane E. Brissenden, Janet M. Roscoe, Mustafa Dogan, Pavlos I. Neophytou, Cemal Gurkan, Ozlem Bulbul, Lotfi Cherni, William C. Speed, Michael Murtha, Judith R. Kidd, Andrew J. Pakstis

**Affiliations:** 1 Department of Genetics, Yale University School of Medicine, 333 Cedar Street, New Haven, CT 06520 USA; 2 Department of Nephrology, Mongolian National University of Medical Sciences, Ulaanbaatar, Mongolia; 3 Independent Scientist, Toronto, ON Canada; 4 Department of Medicine, University of Toronto, Toronto, ON Canada; 5 The Scarborough Hospital, Toronto, ON Canada; 6 Department of Genetics and Bioengineering, International Burch University, Sarajevo, Bosnia and Herzegovina; 7 Mendel Center for Biomedical Sciences, Egkomi, Nicosia Cyprus; 8 Turkish Cypriot DNA Laboratory, Committee On Missing Persons in Cyprus Turkish Cypriot Member Office, Nicosia, North Cyprus Turkey; 9 Dr. Fazıl Küçük Faculty of Medicine, Eastern Mediterranean University, Famagusta, North Cyprus Turkey; 10 Institute of Forensic Science, Istanbul University, Cerrahpasa, 34500 Istanbul, Turkey; 11 Laboratory of Genetics, Immunology and Human Pathologies, Faculty of Sciences of Tunis, University of Tunis El Manar, 2092 Tunis, Tunisia; 12 Higher Institute of Biotechnology of Monastir, Monastir University, 5000 Monastir, Tunisia

**Keywords:** Haplotypes, Population genetics, Genetics

## Abstract

Population genetic studies of North Asian ethnic groups have focused on genetic variation of sex chromosomes and mitochondria. Studies of the extensive variation available from autosomal variation have appeared infrequently. We focus on relationships among population samples using new North Asia microhaplotype data. We combined genotypes from our laboratory on 58 microhaplotypes, distributed across 18 autosomes, on 3945 individuals from 75 populations with corresponding data extracted for 26 populations from the Thousand Genomes consortium and for 22 populations from the GenomeAsia 100 K project. A total of 7107 individuals in 122 total populations are analyzed using STRUCTURE, Principal Component Analysis, and phylogenetic tree analyses. North Asia populations sampled in Mongolia include: Buryats, Mongolians, Altai Kazakhs, and Tsaatans. Available Siberians include samples of Yakut, Khanty, and Komi Zyriane. Analyses of all 122 populations confirm many known relationships and show that most populations from North Asia form a cluster distinct from all other groups. Refinement of analyses on smaller subsets of populations reinforces the distinctiveness of North Asia and shows that the North Asia cluster identifies a region that is ancestral to Native Americans.

## Introduction

Microhaplotypes (microhap, MH) are defined as small genomic regions of less than ~ 300 bp with two or more variants, usually SNPs, resulting in at least three common haplotypes. They were first proposed in 2013^1–3^ as potentially highly informative and useful genetic markers for forensics, anthropology, and biomedical research generally. Since then microhaps have been studied by many forensic researchers with clear demonstration of their potential in such areas as ancestry inference, kinship studies, identifying individuals, detecting mixture samples from multiple individuals, and ecological studies of non-human species. Many of these areas are of great potential interest to physical anthropology. A few papers are starting to use microhaps for anthropology applications: kinship^4,5^ and ancestry^6–9^.

Informativeness (I_n_)^10^ measures the ability of MHs to infer ancestry as has been shown to be quite good for several different panels of MH loci^6,11^, rivaling the Informativeness of the standard forensic STR loci^11,12^. Pakstis et al.^13^ showed that minihaplotypes of 10 kb or smaller were very informative for population differentiation, i.e., ancestry inference of individuals. The same is true for the relatively few studies of microhaplotypes that have been focused on ancestry inference for populations other than those in the 1000 Genomes (1 KG) dataset^14^ such as^6,9,11,15^ although some panels would certainly provide relevant ancestry information^16^ were the analyses done. The general pattern seen in almost all studies is that major continental groups are easily differentiated. In part, this pattern is so distinctive because the sets of populations studied, such as those in the 1000 Genomes (1 KG)^14^, are often clustered geographically with large geographic distances between the clusters.

We have accumulated microhaplotype data on recently available population samples from North Asia, including populations from Mongolia and Siberia (Table 1). These new population samples warrant study for their population relationships using microhaplotype data. The only previous reports of Altai Kazakhs that we are aware of included individuals from various locales in the Russian Altai Republic and those studies were limited to DNA markers on mitochondria^17^ and the Y-chromosome^18^. Other than our studies^2^ we are not aware of any genetic studies of the Komi enclave in N.W. Siberia. (See Compliance with Ethical Standards section for institutional review board details.) Similarly, other than our genetic studies of the Tsaatan^19^ we have found only one study of this population^20^. We have combined data on samples of 75 populations (3945 individuals) from our lab along with data from the 1000 Genomes (1 KG)^14^ and the GenomeAsia 100 K (GA100)^21^ datasets. We examined the biogeographic clustering of 122 population samples while focusing on the North Asia population samples in relation to the existing data on many Eurasian populations and populations from other continents as represented by the specific samples of each. We show that with this set of microhaplotype loci the North Asia populations do not cluster with any of the major biogeographic clusters but have unique global relationships generally unique between the Europeans to the West and the East Asians.

**Table 1:** North Asian population samples studied in the Kidd Lab.

*Kazakh* This sample consists of Kazakh individuals living in Ulgii city in the Bayan-Ulgii aimag of the Altai region of Western Mongolia. This Muslim and Kazakh majority aimag is the westernmost province in Mongolia. This group is also known as the “Eagle Hunters” as depicted in the 2016 movie *The Eagle Huntress*. The individuals speak Kazakh and Mongolian
*Khanty* The sample consists of Khanty individuals living in the Belojarsk area on the River Kazym, a tributary of the Ob River. All individuals sampled and their ancestors were born in this region and belong to the North Khanty sub-ethnic group and the Kazym territorial group. The Khanty language is in the Finno-Ugric branch of the Uralic language family
*Komi* This sample consists of Komi-Zyriane living in the village of Kazak on the Kazak River. The Kazak River is a tributary of the Ob River near the Eastern Ural Mountains. The individuals sampled along with three past generations were born in the taiga area including nearby villages between the Ural Mountains and the Ob River. Individuals of this Komi enclave rely heavily on fishing and hunting and dress in traditional clothing. They speak Komi, a Uralic language
*Mongol* This sample consists of Urban Mongolian individuals living in Ulaanbaatar
*Tsaatan* This sample consists of Tsaatan (Dukha) individuals. The Tsaatan are a small nomadic group of Tyvan-lineage reindeer herders living in northern Khövsgöl aimag of Mongolia near lake Khövsgöl. Their Dukhan language is an endangered Turkic language spoken by only a few hundred individuals; individuals in our sample spoke Mongolian
*Yakut* This sample consists of Yakut-speaking individuals in the Yakut Autonomous Republic of Northeast Siberian Russia. The Yakut language, currently spoken by over 350,000 people, is part of the Turkic branch of the Altaic language family. Individuals sampled were living or were born along the river Lena in the area of Yakutsk and northward, roughly 129-130E, 62-64N

## Material and methods

### Marker methods

#### Microhaplotype selection

Choice of the specific microhaplotypes was determined by maximizing the number of Kidd Lab populations to be studied so that all populations had nearly complete data for all SNPs in all microhaplotypes. Some of the data were included in previous papers^6,22^. All 58 microhaps are among the 198 microhaplotypes with population frequency data in the ALFRED database. Individuals missing genotype data for > 20 (i.e. > 34.5%) of the 58 MH were omitted from the analyses presented here. The full set of microhaplotypes is defined in Supplemental Table S1.

#### Analytic methods

##### Typing methods

All markers typed in the Kidd Lab used TaqMan assays obtained from Thermo Fisher. The individuals with sufficient amounts of DNA available (approximately 50 ng per reaction) were typed following manufacturer’s protocols with reaction volumes reduced to 3 μl, run in 384-well plates, and read on an AB9700HT using Applied Biosystems’ SDS (sequence detection system) software. To maximize the number of SNPs that could be typed on the small amounts of DNA available for some populations, a preamplification protocol was employed as described^19^.

We did not exclude microhaps that showed little variation among the populations previously studied. Table S1 lists the MH loci by their previously published names along with the 171 SNPs used to define the MH alleles in this study. Note that we make a distinction between the general segment of DNA as the named locus and the set of SNPs used to define the alleles at that locus. Use of different sets of SNPs at a locus will result in a different set of haplotypes and their frequencies. The locus name by itself does not carry with it the specific SNPs used to define the alleles. We note, however, that for 56 of those MH included in ALFRED we are using the same SNPs here as in ALFRED; two MH differ—mh11KK-103 and mh16KK-049 (cf. Table S1).

##### Statistical analyses

Haplotypes were inferred using PHASE version 2.1.1^23,24^. Phasing runs only included the individuals with genotype data for all the SNPs defining the particular microhap being analyzed. Since the decision about which individuals to include was made separately for the phasing of each microhaplotype, individuals varied in the number of haplotype genotypes present.

### Population methods

#### Populations with data

The present study involved laboratory genotyping of previously collected DNA samples and then analyses of the data. The samples were anonymized before they were sent to the Yale lab for genotyping. None of the DNA samples was collected for this specific study. No human subject was directly involved in the current research.

As we have been studying microhaplotypes over the past few years, we have accumulated data on samples of many populations. Geographic areas for which we have accumulated data include Siberia and Central Asia. We previously published^19^ data on individual SNPs and some minihaplotypes for a sample of ethnic Mongolians and a sample of the Tsaatan from Northern Mongolia. We have now collected microhaplotype data on samples of six North Asian populations: the Altai Kazakhs from western Mongolia, two other populations from Mongolia (Tsaatan, Mongolians), and three Siberian populations (the Khanty, the Yakut, and an enclave of the Komi Zyriane). We now have data on 58 microhaplotypes for these six North Asia populations and many other populations (Table S1). Table 1 describes the specific North Asian population samples we have studied. Descriptions of the other samples genotyped in this study at Kidd lab can be found in the ALFRED database by using the unique identifiers (UIDs) for these populations which are included in the dataset deposited in the Zenodo archive (See Data availability).

The GenomeAsia 100 K^21^ project has made available whole genome sequence (WGS) data on a sample of 87 Buryats, originating in Mongolia, and on many other populations from Asia. Given our present focus on North Asia, we extracted the GenomeAsia data on Buryats for comparison with our Mongolian and Siberian samples. To provide a global context for these seven North Asia populations we included the data on the populations available in our lab along with the data extracted for these 58 microhaplotypes from 22 of the populations in the GenomeAsia project and from the 26 populations of the 1000 Genomes dataset. In total, we have included data on 122 populations (7107 individuals) for these 58 microhaplotypes. Table 2 lists the populations, abbreviations used, sample sizes, and data sources.

**Table 2:** The list of populations and sample sizes ordered by broad geographic regions.

Region	Source	N	Population	Abrv	Region	Source	N	Population	Abrv
Cen.Africa	KL	69	Biaka	BIA	SoCenAsia	1 KG	102	Telugu	ITU
	KL	38	Mbuti	MBU		KL	30	Keralites	KER
	KL	8	Lisongo	LIS		AG	34	Urban Chennai, India	CNI
W.Africa	1 KG	113	Gambians	GWD		AG	34	Urban Bangalore, India	BGL
	1 KG	85	Mende,SierraLeone	MSL		AG	17	Lambada, India	LMB
	1 KG	99	Esan	ESN		AG	19	Mahar, India	MHR
	1 KG	108	Yoruba, Ibadan	YRI		AG	17	Agharia, India	AGH
	KL	77	Yoruba, Benin City	YOR		AG	20	Toda, India	TOD
	KL	48	Ibo	IBO		KL	13	Thoti	THT
	KL	39	Hausa	HSA		1 KG	102	Tamil, SriLanka	STU
E.Africa	1 KG	99	Luhya, Kenya	LWK		1 KG	86	Bengali, Bangladesh	BEB
	AG	17	Masai, Kenya	MKK		KL	17	Kachari	KCH
	KL	20	Masai, Tanzania	MAS		AG	15	Oraon, India	ORA
	KL	45	Chagga	CGA		AG	17	Konda Reddy, India	KND
	KL	40	Sandawe	SND		AG	20	Birhor, India	BIR
	KL	38	Zaramo	ZRM		AG	15	Onge, India	ONG
Admixed	1 KG	96	Afro-Caribbeans	ACB		AG	15	Hazara, India	HZA
Admixed	1 KG	61	AfrAmer, SW	ASW		AG	20	Mog, India	MOG
Admixed	KL	89	AfrAmericans	AAM	North Asia	KL	45	Khanty	KTY‡
	KL	32	Ethiopians	ETJ		KL	57	Altai Kazakhs	AKZ‡
N.Africa	KL	86	So.Tunisians	TNS		KL	55	Mongolians	OMG‡
S.W.Asia	KL	40	Yemenites	YMJ		KL	43	Tsaatan	TSA‡
	KL	79	Saudi	SAU		AG	87	Buryat	BUR‡
	KL	15	Kuwaiti	KWT		KL	51	Yakut	YAK‡
	KL	62	Palestinian Arabs	PLA	East Asia	KL	54	Koreans	KOR‡
	KL	101	Druze	DRU		AG	150	Koreans	KRE‡
	KL	39	Samaritans	SAM		KL	48	Japanese	JPN‡
	KL	20	Chaldeans	CHL		1 KG,AG	134	Japanese,Tokyo	JPT
	KL	8	Shabaks	SHB		1 KG	103	Han Chinese, Beijing	CHB‡
	KL	117	Syriacs	SYR		1 KG	105	So. Han Chinese	HCS
	KL	140	Yazidi	YZD		KL	48	Chinese, Taiwan	CHT
	KL	133	Kurds	KRD		KL	41	Hakka	HKA
	KL	113	Turkmen	TKM		KL	58	Chinese,SanFrancisco	CHS
	KL	114	Arabs, N.Iraq	NIA		KL	39	Ami	AMI
	KL	39	Iranians	IRN		KL	42	Atayal	ATL
	KL	82	Turkish	TRK		1 KG	93	Dai	CDX
Europe	KL	50	Turkish Cypriots	TCP		1 KG	99	Vietnamese	KHV
	KL	89	Greek Cypriots	GCP		KL	118	Laotians	LAO
	KL	54	Adygei	ADY		KL	24	Cambodians	CBD
	KL	78	Ashkenazi	ASH		KL	11	Malaysians	MLY
	KL	51	Greeks	GRK		AG	25	Austronesians, Indonesia	ASN
	1 KG	107	Tuscans, Italy	TSI		AG	21	Ati, Philippines	ATI
	KL	27	Roman Jews	RMJ		AG	20	Rampasasa,Flores,Indonesia	FLR
	KL	35	Sardinians	SRD		AG	29	Aeta, Philippines	AET
	1 KG	107	Iberians	IBS	Pacific	KL	22	Papuans, New Guinea	PNG
	KL	88	Hungarians	HGR‡		KL	23	Nasioi, Bougainville	NAS
	1 KG	99	N&W Euro.Ancestry	CEU		KL	34	Micronesians	MCR
	KL	85	EuroAmericans	EAM		KL	9	Samoans	SMO
	1 KG	91	Great Britain	GBR	Americas	KL	56	Plains AmerIndians	NPA‡
	KL	114	Irish	IRI‡		KL	51	SW AmerIndians	SWA
	KL	51	Danes	DAN		KL	53	Pima, Mexico	PMM‡
	KL	42	Chuvash	CHV		KL	50	Maya	MAY
	KL	47	Russia,Vologda	RUV		KL	12	Guihiba	GHB
	KL	33	Russians,Archangel	RUA		KL	22	Quechua	QUE
	KL	35	Finns	FIN		1 KG	85	Peruvians	PEL
	1 KG	99	Finns	FN1		KL	65	Ticuna	TIC
W.Siberia	KL	46	Komi Zyriane	KMZ‡		KL	44	Surui,Rondonia	SUR
SoCenAsia	AG	15	Pathans, Pakistan	PHA		KL	55	Karitiana	KAR
	AG	20	Gujjar, Pakistan	GJJ	Admixed	1 KG	64	MexAmr,LosAngeles	MXL
	1 KG	96	Punjabi, Lahore	PJL	Admixed	1 KG	104	Puerto Ricans	PUR
	1 KG	103	Gujarati, Houston	GIH	Admixed	1 KG	94	Colombians	CLM

**‡** = Indicates 15 populations in analyses of North Asia along with outlier groups from nearby regions of the world. **Source**: KL = Kidd Lab, AG = GenomeAsia 100 K project, 1 KG = Thousand Genomes consortium **Abrv**: Three character abbreviation for populations used in various images and tables.

We employed version 2.3.4 of the STRUCTURE software^25^ applying the standard admixture model assuming correlated allele frequencies. The input data consisted of the microhaplotype genotypes for all individuals in the relevant populations. In the STRUCTURE analyses, individuals lacking phased genotypes for more than 20 of the 58 (i.e. > 34.5%) microhaplotypes were excluded from the input files. After censoring, the 122-population dataset included a total of 7107 individuals in the STRUCTURE analyses; 61% of the 7107 individuals have all 58 microhap genotypes present; 90% of the individuals have 52 or more genotypes. The program was run 20 times at each K level from K = 2 to K = 11 with 10,000 burn-in and 10,000 Markov Chain Monte Carlo (MCMC) iterations.

For the Principal Component Analyses (PCA) we used the XLSTAT 2019 software (http://www.xlstat.com/en/about-us/company.html) on the matrix of haplotype allele frequencies for all 58 microhaplotype loci and the populations relevant to each analysis.

Phylogenetic population trees for the 74 and 15 population datasets were generated using a Least Squares solution for an additive model. We calculated tau genetic distances^26^ and generated a tree using the Neighbor Joining (NJ) utility that is part of the PHYLIP software package^27,28^. A NJ tree gives an approximate Least Squares fit of an additive tree structure to pairwise genetic distances. This tree was then evaluated by exact Least Squares and many possible trees were examined for their fit to the additive model using a heuristic method of successively altering the tree structure^29^. The Drawtree utility (version 3.69) in PHYLIP was employed to plot the postscript images of the best population trees.

### Ethical approval

All subjects gave permission for collection of samples and for their use in general population studies of which this is one. The present study involved laboratory genotyping of previously collected DNA samples and then analyses of the data. The samples were anonymized before they were sent to the Yale lab for genotyping. None of the DNA samples was collected for this specific study. No human subject was directly involved in the current research. All samples in this study are anonymous and therefore this study, per se, is not considered human subjects research by NIH and by Yale University review committee. The various population samples studied in the Kidd laboratory since 1985 have all been collected with informed consent under a general Yale University protocol (HIC#8,711,001,387) which was also reviewed and approved by the NIGMS (National Institute of General Medical Sciences of the U.S. National Institute of Health) and by CEPH (Center for the Study of Human Polymorphisms in Paris) as well. One third of the samples in the CEPH-HGDP (Human Genome Diversity Project.) collection came from the Kidd lab. In this report the newly collected Altai Kazakhs are the only population sample not previously included in any publication. Signed informed consent for the Altai Kazakh individuals who volunteered to give saliva samples for population studies was obtained by Dr. Ariunaa Togtokh, (cf. co-author affiliation) in Mongolia and Dr. Janet Roscoe, (cf. co-author affiliation) in Canada. The samples were collected under protocols approved by the Ethics Review Board of the Ministry of Health in Ulaanbaatar, Mongolia and by The Scarborough Hospital Research Ethics Board in Scarborough, Ontario, Canada.

## Results

Data for 22 populations (657 individuals) were extracted from the GenomeAsia 100 K database for the SNPs in these 58 microhaps (Table S1). Data for the 122 populations listed in Table 2 also includes the 26 populations extracted from the 1000 Genomes project website. These data were integrated with the TaqMan data generated in our lab for the other 75 populations in the study. The individual genotypes for all 58 microhaps for those 75 populations have been deposited in the Zenodo archive (See Data availability section). Data for the other populations came from the 1 KG or GenomeAsia database. The ALFRED frequency database (https://alfred.med.yale.edu) does contain the allele frequencies for 56 of the 58 microhaps for 91 of the 122 populations in this report, including some of the 1 KG populations. ALFRED is freely accessible online but has been static since 2019; thus, the frequencies for the 31 newest populations, especially those from the GenomeAsia database, are not available in ALFRED.

Hardy–Weinberg ratios. The observed and expected genotype counts for each of the 171 SNPs defining the microhaps studied in the 75 Kidd lab population samples were compared for goodness of fit to Hardy–Weinberg ratios via a likelihood-ratio chi-square test. The deviations from Hardy–Weinberg ratios detected at nominal thresholds (such as 5% or 1%) were those one expects to find by chance when conducting a large number of tests. A total of 11,921 out of the 12,825 theoretically possible tests (171 SNPs × 75 populations) could be carried out; when a single fixed genotype occurs for a particular SNP by population combination, there is nothing to test.

Phasing Accuracy and Data Completeness. Genotypes were assigned with the PHASE program for individuals having SNP genotypes for all the defining SNPs of a microhap. When the probability of phasing fell below 0.98, the assignments were checked to determine whether the genotype should be omitted. In the current dataset, these follow-up checks typically involved rare and very low frequency haplotypes occurring in the Sub-Saharan African population samples and the genotypes were not omitted. For the 7107 individuals analyzed in the 122- population dataset, 3.17% of the 411,800 possible MH genotypes were missing. The missing genotype rate was similar at 3.20% for the 15-population subset and is only 1.66% for the 74- population subset. PHASE software yields a statistically accurate estimate of allele frequencies for populations and provides maximum likelihood estimates of the haplotype genotypes of individuals when each SNP is typed individually^30^.

Locus characteristics. The populations in this study show high average heterozygosity for the 58 microhaps. The average A_e_ value is 2.72 in the dataset of 122 populations; this is equivalent to an average heterozygosity of 0.632. Thus, these 58 loci are, on average, more heterozygous than is possible for 58 di-allelic SNPs. The frequency distribution of A_e_ values is shown in Fig. S1.

**Figure 1:**
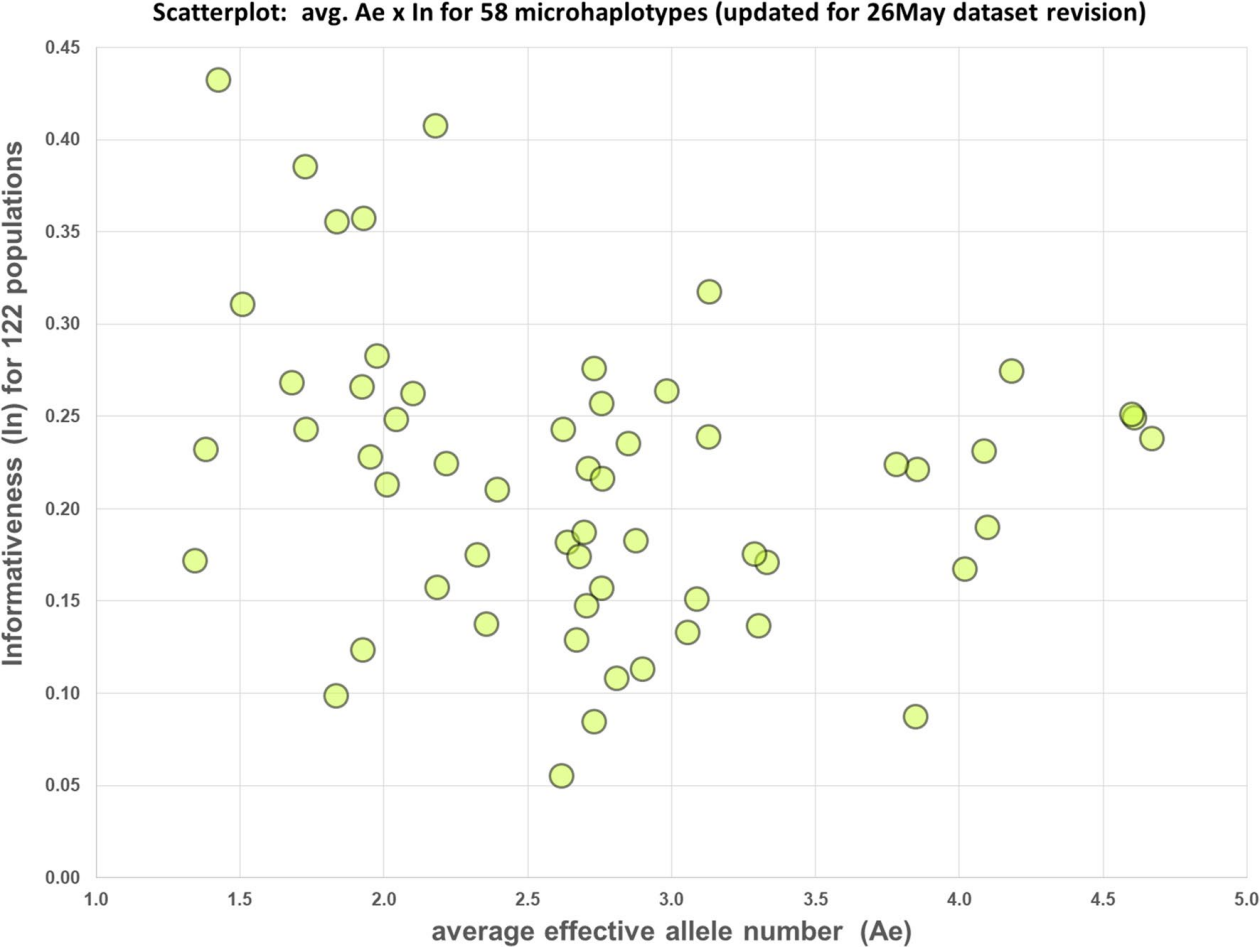
Scatterplot of A_e_ by I_n_ for 58 microhaps evaluated on 122 populations. See text.

The average and median informativeness (I_n_) values, 0.215 and 0.221, respectively, differ little. The I_n_ values range from 0.055 to 0.432. Figure 1 presents a scatter plot of the average A_e_ and the I_n_ values for each of the 58 microhaps. Strong differentiation (I_n_ > 0.2) among the populations studied occurred across the full range of average A_e_ values. However, 4 of the 58 microhaps only differentiated weakly (with I_n_ < 0.10) among the 122 populations and so they were not contributing very much to population differentiation in the PCA and STRUCTURE results.

### 122-population analyses (7107 individuals)

**Figure 2:**
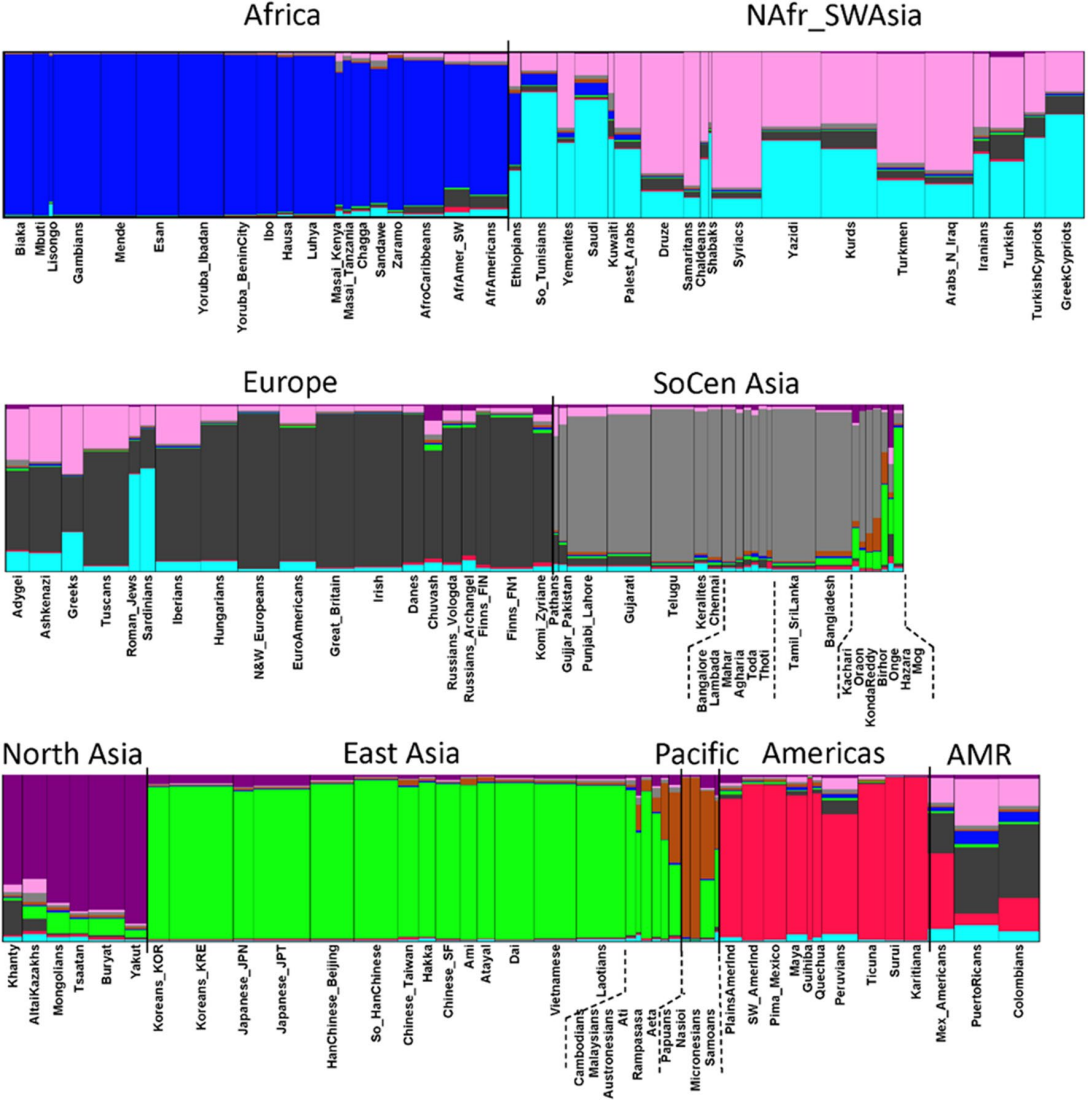
STRUCTURE results by population averages for 122 populations at K = 9. The result with the highest likelihood is shown as population averages. AMR indicates the American populations from the 1000 Genomes dataset that have very low Native American ancestry and have predominantly European and some West African ancestry. The plots for K = 7 through 9 are shown in Supplemental Fig. S2; plots of likelihoods for K values are in Supplemental Fig. S5.

STRUCTURE analyses that included all 122 populations are shown as population averages for K = 9 values in Fig. 2 and also for K = 7 through 9 in Fig. S2. At K = 7 the results divided these samples of populations into broad geographic groups: sub-Saharan Africa, Southwest Asia—North Africa—Mediterranean Europe, Northern Europe, South Central Asia, East Asia, the Pacific, and the Americas. The highly admixed American populations from the 1 KG showed substantial European cluster assignments. The Peruvian sample from the 1 KG showed more admixture than other Native American populations but clustered with them. The sample of Komi Zyriane from Northwestern Siberia clustered with the Northern Europeans while the rest of the North Asian samples clustered primarily with East Asian population samples but did show a small fractional assignment to the Northern European cluster. By K = 8 the individuals from Southern Europe and Southwest Asia and North Africa were attributed to three clusters with no clear internal geographic clustering into populations. Northern European populations (with the Komi) form a cluster with few partial assignments to the clusters so prominent in the other circum-Mediterranean and southwest Asian populations. K = 9 is the first level at which the North Asian population samples (minus the Komi) form a distinct cluster.

**Figure 3:**
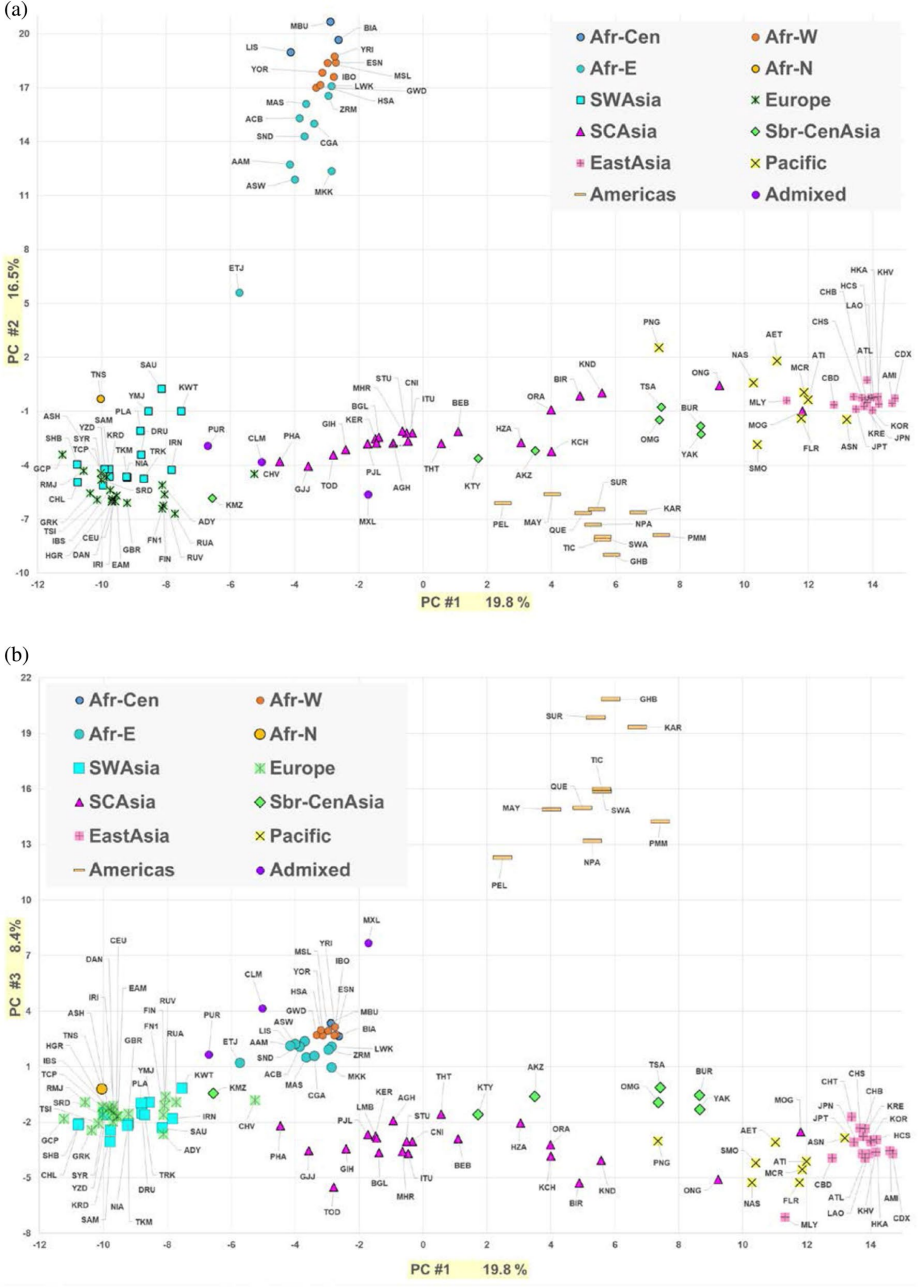
PCA results for 122 populations. (**a**) PC1 by PC2. (**b**) PC1 by PC3.

PCA analyses of the 122-population dataset (Fig. 3) show PC1 (19.8%) with the Europeans at one end and the East Asians at the other; the remaining populations were distributed between these extremes. PC2 (16.5%) separated the populations from Africa from all the rest and to a smaller degree clustered the Native Americans at the opposite end of the axis. The distinctiveness of most Native Americans was emphasized at PC3 (8.4%; Fig. 3b). The highly admixed Americans from the 1 KG (Colombians, Puerto Ricans, Mexican Americans) clustered closer to the European samples. The Peruvians from the 1 KG clustered closer to the other Native American samples, consistent with less European admixture in this 1 KG sample.

These PCA and STRUCTURE analyses of all 122 populations with data for these 58 microhaps showed that these sub-Saharan African populations were very different from non-African populations, in agreement with the consensus on the out of Africa model of modern human history^31^. A “cloud” of the populations from Southwest Asia, North Africa and Southern Europe suggested complex relationships which have been shown in analyses focused on most of those populations but based on various sets of SNPs^32,33^ and microhaplotypes^22^. These populations present a complex set of relationships and a new analysis of relationships among these populations awaits assembly of additional data.

Given our present focus on North Asia, we decided, based on these STRUCTURE and PCA analyses, to omit African, Southern European, Southwest Asian and the three 1 KG admixed American populations from additional analyses. This will focus analyses of the reduced dataset of 74 populations on the allele frequency differences among these populations, but still employing all 58 microhaps, as described in the next section.

### 74-population analyses (3838 individuals)

**Figure 4:**
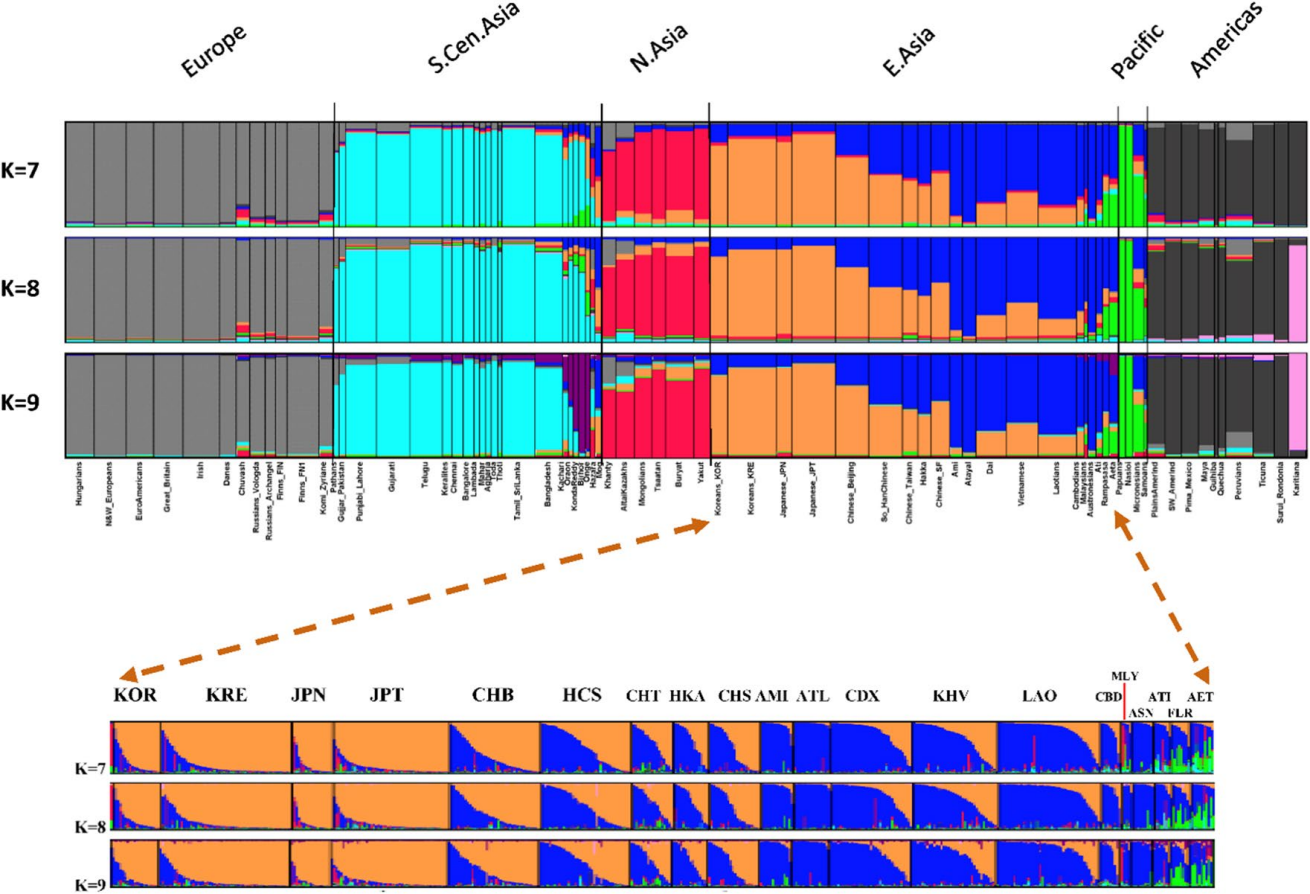
STRUCTURE results by population averages for 74 populations at K = 7 through K = 9. At each K value the result with the highest likelihood is shown as population average. This subset of the 122 populations is in the same order as in Table 2. The plots of individuals for these three K values are shown in Supplemental Fig. S4; plots of likelihoods for K values are in Supplemental Fig. S6. In the lower part of the figure, individual bar plots are shown for East Asia with the individuals sorted by the major cluster values within each population.

STRUCTURE analyses of the 74-population dataset are shown in Fig. 4 as population averages for the most relevant K values for our focus on North Asia. At lower K values the regional populations became distinct (see Fig. S3). At K = 4 only one pattern was present in all 20 runs: Northern Europe, South Asia, then North Asia, East Asia, and Pacific together, and the Americas. At this and all higher K values the Komi always clustered with the Northern Europeans. At K = 5 and K = 6, the patterns with the highest likelihood had the Pacific populations as a distinct cluster, although at both K values the second most common pattern had the North Asians as a different cluster. In both cases several runs produced alternate cluster patterns that had similarity measures > 0.95. By K = 7 (Fig. 4) the North Asian population samples consistently formed a distinct cluster at the various solutions. At K = 7 through K = 9 the most likely patterns had the Khanty showing heterogeneity with some similarity to both the European and Central Asian clusters, and the remaining Northern Asian samples forming a distinct cluster. We note that at these K values the 58 microhaps showed most East Asian populations fall into two clusters with considerable heterogeneity among individuals. The two Korean samples and the two Japanese samples formed a more distinct cluster that has little similarity to the more heterogeneous cluster pattern seen elsewhere in East Asia for the many Chinese and Southeast Asian samples. In the lower part of Fig. 4 individual bar plots are shown for East Asia with the individuals sorted by the major cluster values. By K = 9 several of the populations from India with small sample sizes formed a distinct cluster. These populations are described and discussed in the GenomeAsia paper^21^.

**Figure 5:**
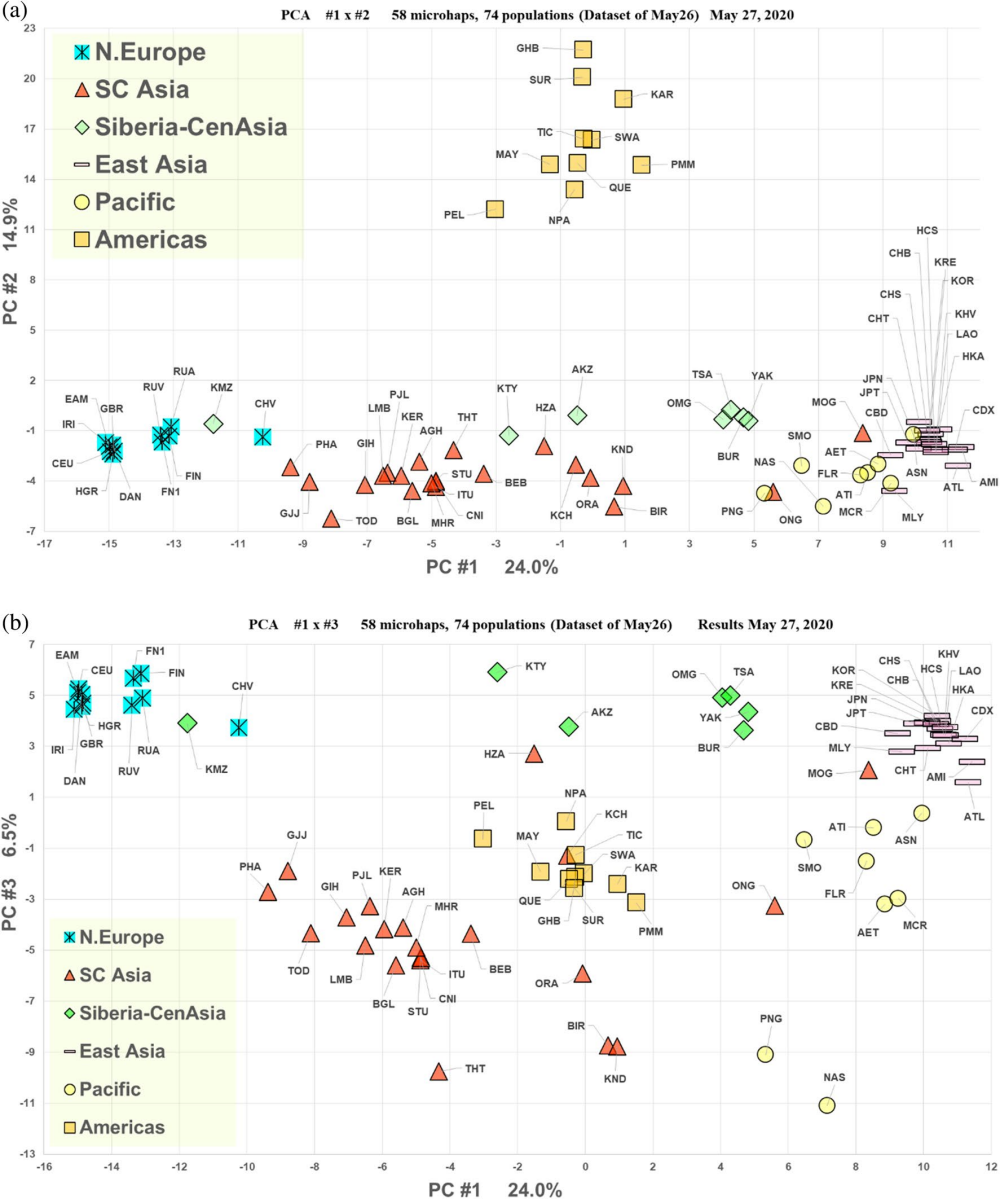
PCA results for 74 populations. (**a**) PC1 by PC2. (**b**) PC1 by PC3.

The PCA analysis of the same 74-population dataset (Fig. 5) showed a pattern largely consistent with the STRUCTURE analyses. PC1 (24.0% of the variance) had the Europeans and East Asians positioned at opposite extremes. The South Asian populations were distributed across the distance between those extremes. The Native Americans were completely distinct based on PC2 (14.9%) while there was little difference on that axis among the remaining populations. The Komi sample clustered adjacent to the Northeast Europeans. The Khanty and Altai Kazakhs were plotted among the South Asians. The other four North Asians were tightly clustered and distinct from the East Asians. When considering PC3 (6.5%) in Fig. 5b all six North Asians were distinct from the other Asians though still aligned on PC3 with the Europeans. The Pacific populations were distinct from East Asian populations on PC3. In contrast to the STRUCTURE results the Northeast Europeans separated slightly from the other European populations. Similarly, the Khanty were intermediate between the main North Asia cluster and the Northeast Europeans.

**Figure 6:**
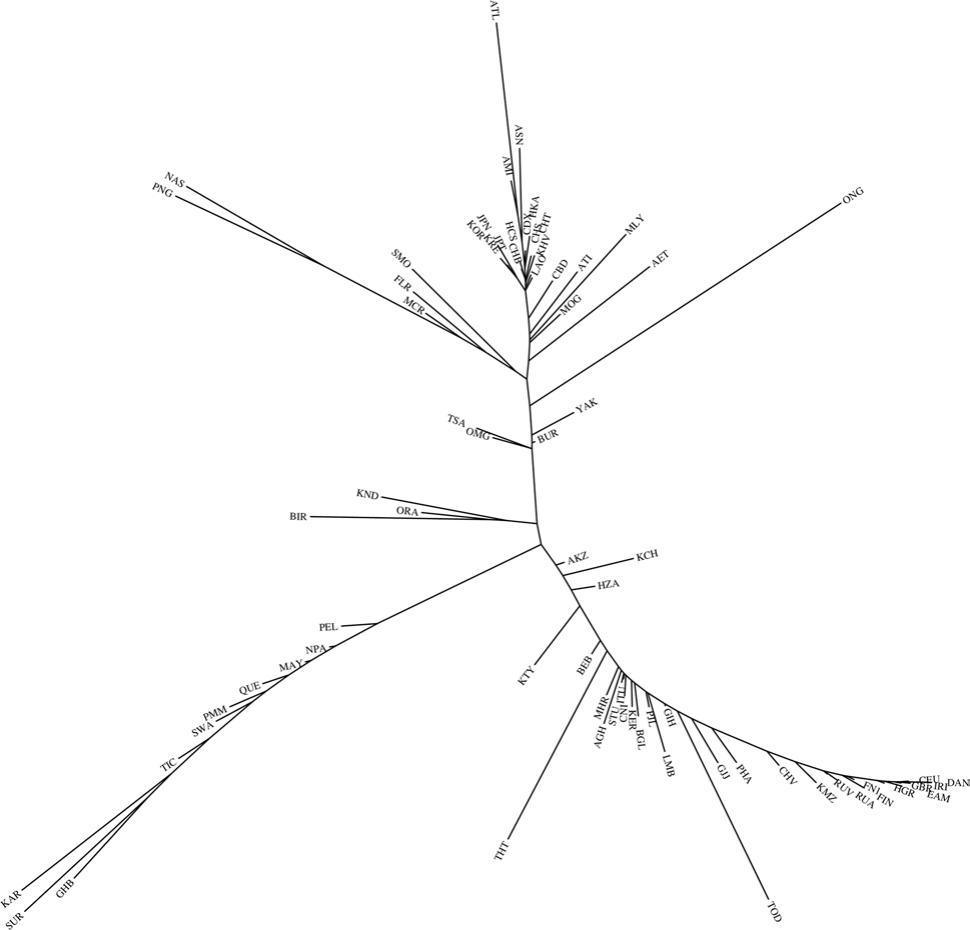
Best exact Least Squares tree for 74 populations. This best of 445 different trees examined has no internal negative segments but does have eight external negative segments to the CHV, FIN1, GIH, PJL, BEB, PEL, BUR, and JPT populations. In their local positions in the graph the segments are small in absolute value and are indicative of small deviations from true additivity in their pairwise genetic distances. They generally are located in reasonable locations with respect to what is known about their relationships. For example, the FIN1 sample from 1 KG clusters with the independent FIN sample from Kidd Lab.

We examined by an exact least-squares fit to the tau distance matrix a total of 445 different additive tree structures (including the Neighbor Joining tree). Of those, 35 had no internal negative segments and the best of them is illustrated in Fig. 6. We are reluctant to place great reliance on any specific fine structure because so many of the populations likely deviate significantly from the assumption of additive distances. Nonetheless, some aspects are consistent among the several better trees and past tree analyses of independent genetic markers^34^. The East Asian populations clustered close together on small branches arguing that this set of microhaplotypes was not very informative for distinguishing East Asian populations from each other. Similarly, Western European populations were very close together. The Native Americans were distributed on a long branch connecting to the “middle” of the tree surrounded by the North Asia populations, the three Mongolian populations and Yakut on the East Asian side and the Altai Kazakhs and Khanty on the South Asia-Europe side. Several populations were consistently placed on long terminal branches, indicating they had allele frequencies considerably different from the other populations.

The pattern in these 74-population analyses for the North Asian populations was for the Komi to cluster with the other Europeans, the Khanty to show attribution to both the European and the North Asian clusters, and the remaining populations to be primarily allocated to a distinct North Asia cluster. Given these results, we have focused our additional analyses on the allele frequency variation in just a subset of 15 populations: the seven North Asian populations, the Irish and Hungarians on the European side, the two Korean samples, the Kidd Lab Japanese, and the Beijing Han on the East Asian side, and the Plains American Indians and Mexican Pima in the New World.

### 15-population analyses (1050 individuals)

**Figure 7:**
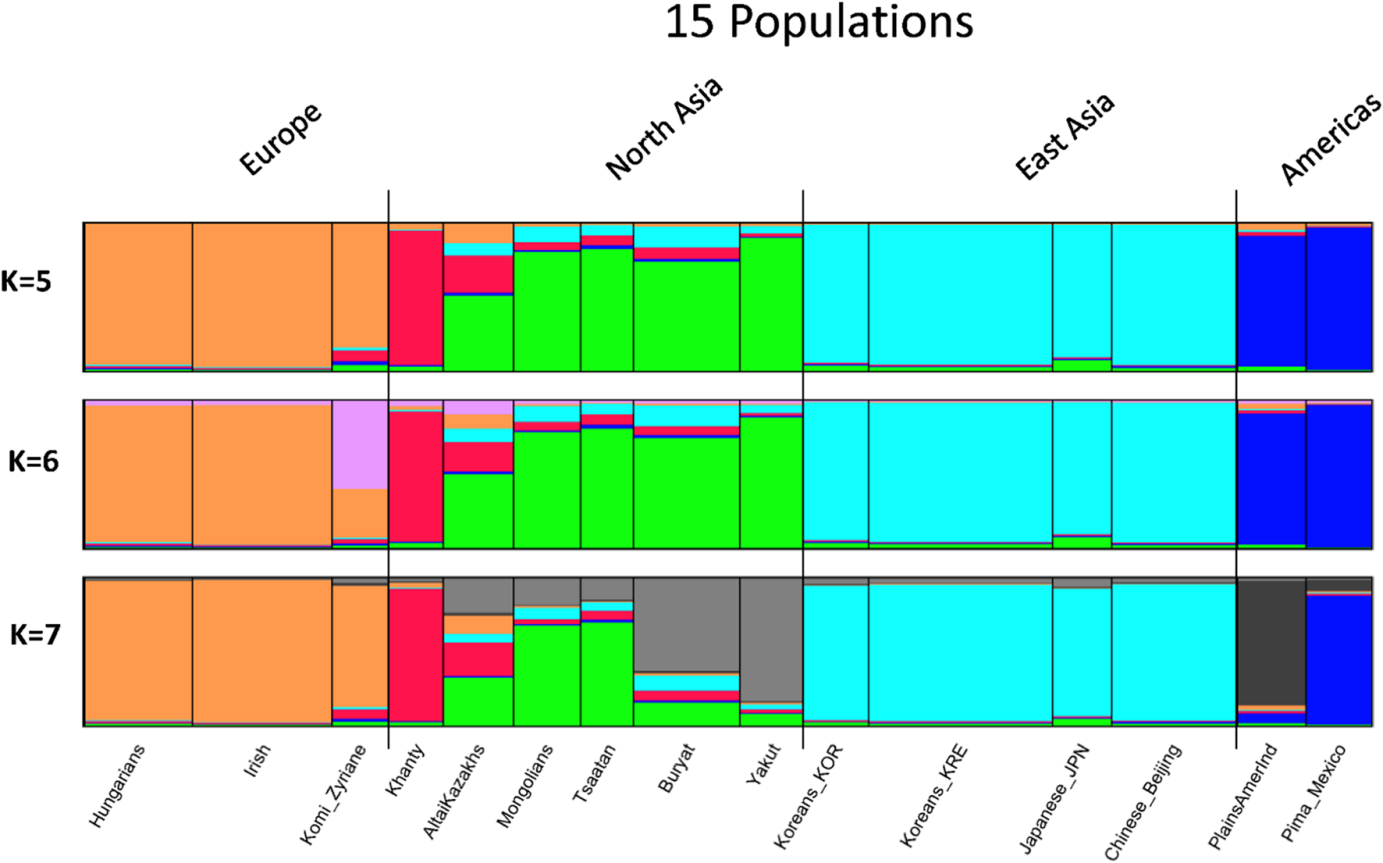
STRUCTURE results by population averages for 15 populations at K = 5 through K = 7. At each K value the result with the highest likelihood is shown as population averages. The plots of individuals for these three K values are shown in Supplemental Fig. S6; plots of likelihoods for K values are in Supplemental Fig. S8.

The STRUCTURE analysis of those 15 populations (Fig. 7) showed them to be gradually sub-dividing with increasing K values such that the cluster pattern went from four “regional” groups (Europe, North Asia, East Asia, Americas) to six groups with separation of the Komi and Khanty into different groups. At K = 5 the Khanty become a distinct cluster. At K = 6 the best result had a partial assignment of the Komi to a new cluster. At K = 7 the five remaining North Asians showed considerable individual heterogeneity with the Yakut showing the least. The Komi were no longer distinct from the other European populations but the two Native American populations were distinct. Among the North Asians the Buryat and Yakut populations seemed the most similar. Increasing K values resulted in great individual heterogeneity among the four East Asian populations.

**Figure 8:**
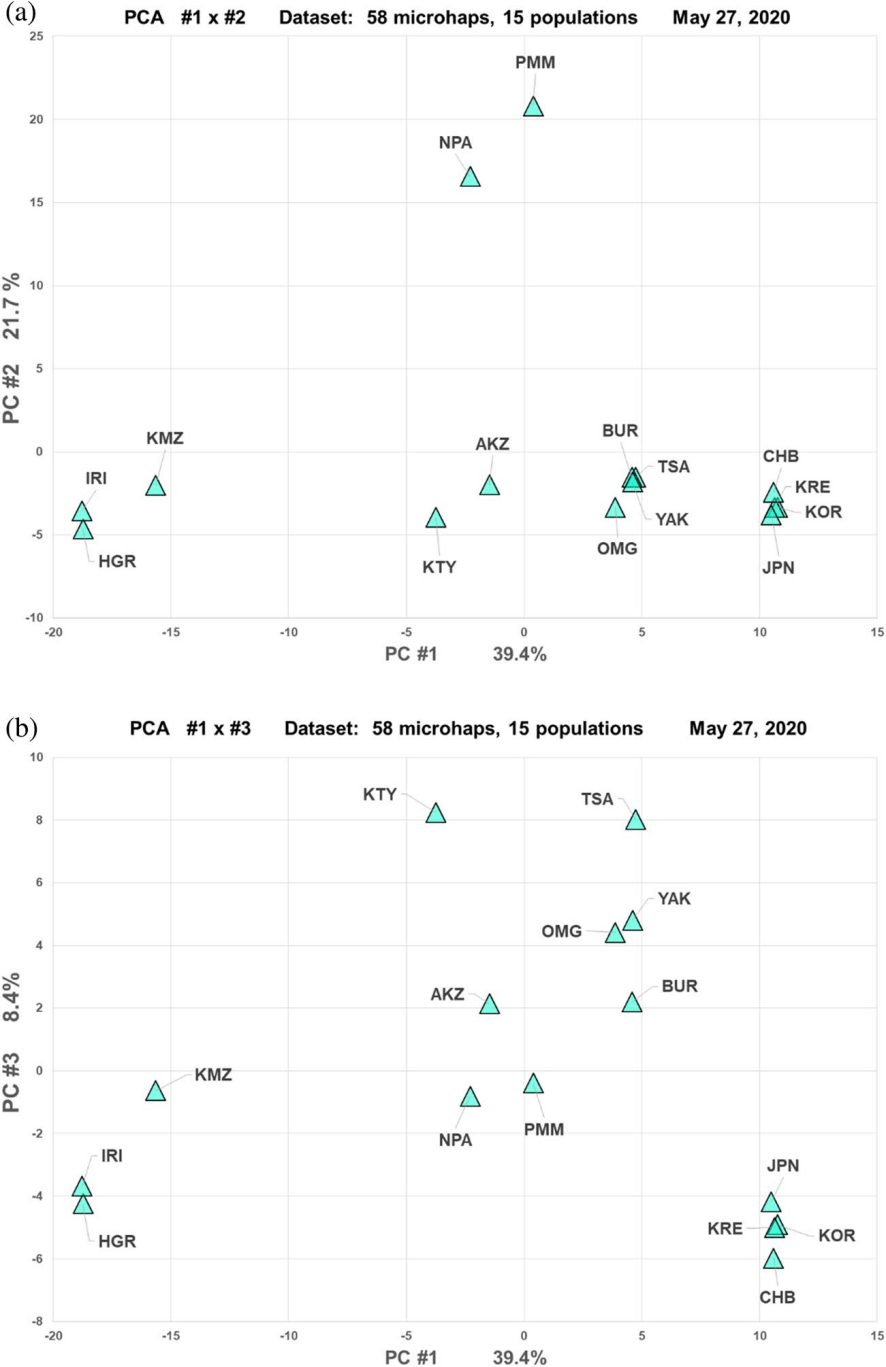
PCA results for 15 populations. (**a**) PC1 by PC2. (**b**) PC1 by PC3.

The PCA results on the subset of 15 populations (Fig. 8) show, as expected, that PC#1 (39.4% of the variance) positioned the European and East Asian populations at opposite extremes. PC#2 (21.7%) primarily separated the two Native American populations from the other populations; except for the Komi, the North Asians were, on average, closer to the East Asians than to the Europeans. For PC#3 (8.4%) in Fig. 8b the Khanty compared to the East Asian populations defined the extremes of the axis. The East Asians were tightly clustered and were distinct on PC3 from the North Asians. The first three PC’s gave little indication of a linear relationship among the 7 North Asian populations that would correspond with the Great Circle distances across the longitudes.

Including the Neighbor Joining tree, 54 different exact Least Squares trees were examined for the 15 populations (Fig. 7). Of these, seven trees had no negative internal segments. The best of these seven had small negative external segments for the Khanty, the Altai Kazakhs, and Buryats. This tree had the same basic pattern of population relationships as the corresponding populations in the 74-population tree. The main axis of the best tree also corresponded closely to the distribution of populations on PC1 in Fig. 3a with Europeans and East Asians forming flanking clusters at opposite ends of PC1.

## Discussion

**Figure 9:**
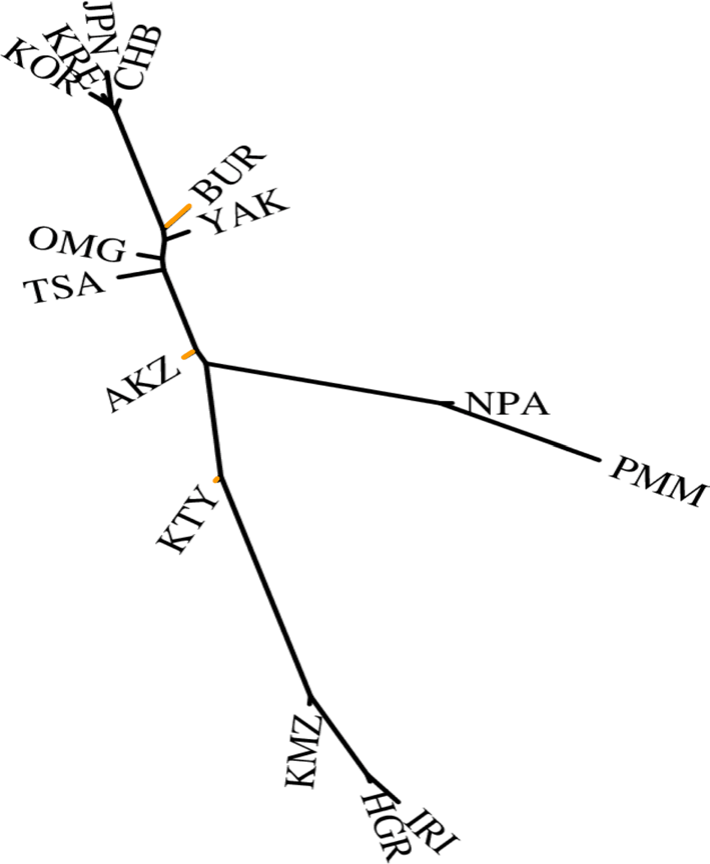
Best exact Least Squares tree for 15 populations. Negative branches to the Buryat, Altai Kazakhs, and Khanty are orange.

Most of the relationships indicated by our analyses of the population samples we have available are noted in Results. These analyses confirm with microhaplotypes many previous studies with other types of genetic markers and the various anthropologic studies of the populations across Eurasia^35,36^. Those relationships show a West to East cline in genetic similarity and distinction of the Siberian populations from the Europeans to the West (typified by Irish and Russians) and Asians to the East (typified by Koreans, Japanese, and Han Chinese). Also, our results show, as other studies have shown, that those major East Asian populations form a very tight cluster^6,19,21^. Our analyses show that the major East Asian populations are, as a cluster, very distinct from the North Asia samples (e.g., Figs. 8 and 9).

The North Asia population samples we have studied generally show different patterns of relationship depending on the method of analysis and set of populations considered. The one consistent result is for the Komi. The Komi sample we have studied is part of a Northwestern Siberia enclave of what is essentially a European population. Our sample of the Komi is geographically relatively close to our sample of the Northern Khanty but genetically they are proportionately much more distant. In Fig. 5 we see the Komi and Chuvash loosely clustered with the Central Europe and Northern Europe populations. The Komi are as different from the Finns and Russians as those are from the Central Europeans. The Finns and Russians are tightly clustered together even though they speak languages from different linguistic families: Uralic for the Finns and Slavic for the Russians. A genetic difference between the Komi and Khanty agrees with the linguistic tree of Tambets et al.^37^ that has the Ugric-Khanty and Permic-Komi branches of the Uralic speaking populations on different major linguistic branches. The Y and mtDNA patterns are also different with more Eastern Eurasian variation in the Khanty than in the Komi but the Tambets et al.^37^ sample of the Komi is different from ours making it hard to compare the results. Their large number of autosomal SNPs studied (hundreds of thousands) showed that, using ADMIXTURE software, the Uralic speakers resembled their neighbors rather than forming a cluster unto themselves. Based on STRUCTURE our Komi sample from Western Siberia was predominantly part of the European cluster until K = 6 using the highest likelihood result for the 74 population analyses. At the higher K values the Komi remained distinct from the European populations but there was still considerable attribution to the European cluster for many individuals. The conclusion of both PCA and STRUCTURE analyses of the Komi is consistent with the larger Komi ethnic group being essentially a European population spanning the northern boundary between Europe and Asia.

The Khanty sample does not have a simple genetic interpretation in terms of possible origins since it shows similarity to both European and more eastern Asian populations; it is intermediate in one sense (cf. Figs. 3 and 5) but as seen in several of the analyses it is also unique (e.g., Figs. 7 and 9). In the Tambets et al.^37^ analyses the Khanty were in a different cluster than the Komi. The Khanty were somewhat intermediate between the Komi and the Yakut. Greater density of population samples studied for these autosomal markers will be necessary to integrate the Khanty into the genetic pattern of Siberian populations. A similar comment is warranted for the Altai Kazakhs. This sample clusters with the other North Asian samples using STRUCTURE (cf. Figure 7) but with PCA and tree analyses is also distinct from the Buryat, Tsaatan, Mongolian, and Yakut samples (cf. Figs. 5, 8, and 9). Lacking other relevant samples, such as Kazakhs from Kazakhstan, Kyrgyz, and Uzbeks, it is not possible to fully understand the full pattern of variation in North and Central Asia. The Altai show evidence of ancient mtDNA reflecting the ancestry of the current population^38^. The mtDNA gene pool of over 200 Altai Kazakh individuals showed both West and East Eurasian haplogroups^17^. The whole genome sequence of a Kazakh individual shows similarity with other Kazakh and Buryat samples as intermediate between European and East Asian samples^39^. Y chromosome studies of the Altai Kazakhs suggest they are part of the general Kazakh ethnic groups but with some influence from the thirteenth century Mongolian empire. To the degree that the population samples from Jeong et al.^35^ and Fedorova et al.^40^ overlap with our samples, the SNP-based relationships agree with the microhaplotype-based relationships.

Our samples of Khanty and Altai Kazakhs are distinct from each other and the Mongolians by PCA (Figs. 3 and 5) but cluster with the Mongolian and Yakut samples by STRUCTURE (Figs. 2 and 4). The samples are large enough that a strong underlying genetic similarity must exist for the six groups to cluster together by STRUCTURE. The clustering is different when only a few populations are included (Fig. 7). This illustrates the dependence of STRUCTURE analyses of some populations on the context of “reference” populations. Whether different loci may be influencing the analyses differently is a possibility for future analyses to explore. However, that a difference is seen for a set of autosomal loci is a cautionary note on interpretations of population similarities based on a single analysis or a single type of analysis.

The three populations sampled in Mongolia cluster together by both STRUCTURE and PCA and are very close in the tree analyses. mtDNA and Y-chromosome variation also argue that the Buryat are similar to the Tsaatan and Ulaanbaatar Mongolian samples. The Yakut also cluster with the Mongolian pops in agreement with the many studies showing close linguistic and genetic similarities to populations in the Lake Baikal region. One aspect of the pattern is that once STRUCTURE reaches a finer level of resolution (K = 9) the PCA and tree analyses agree that the Buryat, Yakut, and both Mongolian samples form a clear cluster of closely related populations. For example, Kilinc et al.^41^ noted “… little is known about the population history in North Asia.” They then examined many ancient mtDNA samples and concluded the data suggest long term maternal stability in the region from Lake Baikal to Yakutia. Triska et al.^42^ found with autosomal SNPs that their Yakut and Buryat samples were more similar than the regression of genetic similarity and geography would predict. Our results confirm a clear genetic similarity despite a current geographic separation. A recent origin of the Yakut from a more southern and central Asian region near Lake Baikal is the general consensus also based on mtDNA and Y-chromosome variation^40,43^.

Liu et al.^44^ fit to the HGDP dataset^45^ a model of successive founder effects as modern humans spread from origins in Africa to occupy the rest of Eurasia. The result is consistent with the cline from West to East of loss of heterozygosity and increasing genetic distance correlated with distance from human origins in Africa^46^. As seen in, e.g., Fig. 5, the first principal component axis shows the North Asia populations distributed from West to East. That ancient cline has undoubtedly been modified by recent migrations and admixture. Other than the relatively recent migration of the Yakut what effects other migrations may have had on our specific population samples is unclear.

Bai et al.^47^ carried out whole genome sequencing on 175 ethnic Mongolians to improve genetic sampling of Northern Asia. Five of the tribal/location samples out of six groups they studied were from Inner Mongolia, China. One sample of Khalkha was from Ulaanbaatar. Their six Mongolian population samples clustered with the East Asian populations using ADMIXTURE until, at K = 9 and K = 10, they became a distinct cluster. Their maximum likelihood tree includes three of their Mongolian populations and places them at the base of the branch into East and Southeast Asia. The selection of ethnic population samples by Bai et al. is sufficiently different that detailed comparisons are not possible. To the degree comparison is possible the results are compatible with our results.

The three different methods used in this study provide different views of the data based on different statistics with different underlying assumptions. The STRUCTURE software groups individuals to form Mendelian populations, i.e., K-clusters of individuals each of which approximates a population showing Hardy–Weinberg ratios of the genotypes. When an individual does not fit well into one of the K populations, it can be assigned partial membership in different “ancestral” populations. This can be considered admixture but could also just reflect the intermediate nature of an individual or population given the fixed number of K clusters allowed. This distinction between “admixed” and “distinct” clusters can be seen for the North Asians in Fig. S2 comparing results for K = 8 and K = 9. The converse is seen in the same Figure between K = 7 and K = 8 when the freedom of an additional K value allows more variation to become evident.

We emphasize that the STRUCTURE analyses are done by individual microhap genotype profiles and our representations in the Figures have the individuals grouped by population (Supplemental Figs. S2, S4, and S6). The correspondence of cluster assignments of individuals to the grouping by populations validates the definitions of the population samples. However, there need to be enough individuals with a distinct genotype array to generate a valid cluster at a specified K value. A small sample with distinct allele frequencies can be absorbed into a larger cluster without greatly altering the Hardy–Weinberg distribution. Strictly speaking, a STRUCTURE analysis provides no information on how the different clusters are related to each other. A series of analyses at different K values may convey some hierarchical information as clusters at lower K values are subdivided at higher K values. This is illustrated in Fig. 4 with the Karitiana separated at higher K values from the other Native American population samples.

PCA plots show graphically the similarities and differences in the elements of the input data. If the data are organized by individual, a population will be shown as a cloud of points representing the inherent differences among individuals within a population. The closeness of the clouds for different populations, even the overlap of those clouds, can be quantified as the distances on one or more of the axes. Because we are analyzing multiallelic data, we utilized the population allele (haplotype) frequencies rather than the individual genotypes and as a result have plots of points representing the population samples. The points along the orthogonal axes represent the centroids of the individuals assigned to the population. We note that, as in STRUCTURE analyses, the specific populations included in PCA can alter the relationships shown for other populations. Thus, the relationships of the populations in the 15-population analysis (Fig. 7) are somewhat different than in the 74-population analysis (Figs. 2 and 4).

A common form of tree analysis in the literature is the Neighbor Joining (NJ) tree^48^. An NJ tree is an approximate additive tree, meaning that the pairwise distances between populations are apportioned so that they are additive across all the tree segments between the population pairs. The statistic approximated is a least-squares fit to the series of equations representing the tree structure^29^. An exact least-squares (LS) fit to a distance matrix is a reasonable representation of the data to the degree that the assumption of an additive distance between populations is met. Consistent with that assumption, we have used a genetic distance measure that estimates the pairwise distance in units of t/2N_e_, i.e., time in generations divided by twice the effective population size. This assumes random genetic drift is the cause of the differences among populations. This distance measure is then additive across the correct tree structure with the objective being to identify the best fitting tree structure. As noted in METHODS, an iterative approach was used to find the best fitting LS tree structures. Because of the very large number of tree structures possible for any large set of populations, it is convenient to start with the NJ tree which is usually similar to the better LS trees. A major difference in our experience is that the NJ tree typically has many negative segments while the best LS tree has fewer or none. A negative segment clearly identifies a region of the tree that violates the additivity assumption.

Although our focus in this study was on North Asia, other interesting findings emerged in our analyses of the large amount of information. We are not giving them much attention because additional data will likely become available for many of these other populations and more certainty will then be possible with more data. However, these analyses do support some conclusions on other populations beyond the North Asia populations. The most distinctive of these interesting other findings is support for the ancestry of the Native Americans originating from the middle of North Asia. This is not a new conclusion. Studies of Y-chromosomes argued for the Altai region of Siberia as the origin of Amerind speaking Native Americans^49,50^. An independent study of Y chromosome variation and mitochondrial DNA variation^51^ reached essentially the same conclusion that southern Altaians are the closest to the ancestors of Native Americans. Other studies including Northern American Natives (e.g., Eskimos)^52^ argued for a more eastern Siberian origin of those populations. Our studies are limited to those Native Americans classified as Amerind speaking.

Our current study and our previous reports^19,34,53^ provide extensive independent autosomal data. The STRUCTURE analyses (Figs. 2 and 4) show the distinctiveness of the Native Americans but cannot readily show the ancestral relationships of that cluster to the other population clusters. The PCA analyses provide some information in that in both Figs. 3 and 5 the Native Americans fall mid-way along the PC1 axis and as distinct on PC2. The tree analyses (Figs. 6 and 9) provide an example of autosomal genetic data placing the origin of the Native American populations in the middle of Siberia. The interpretation of the tree diagrams is that pairwise F_st_ values between Native American and other populations are smallest with the North Asian populations. Previous tree analyses with independent autosomal data have shown a similar placement of the base of the Native American branch^19,34^. We note that the base of the Native American branch is long, indicating considerable genetic drift in common to all extant Native American populations sampled, i.e., its length reflects the genetic bottleneck in the initial colonization of the Americas.

At higher K values the STRUCTURE algorithm is basing its clusters on smaller subsets of the allele frequency data and smaller differences in allele frequencies. We do note that the heterogeneity is largely on an individual by individual basis. At K = 7–9 the samples of East Asian populations showed considerable variation by individual. The population averages fail to reveal this. The population averages do give a hint that the patterns of variation are different among the populations with some individuals belonging primarily to one cluster and other individuals primarily to the other cluster. When plotted by individual and the individuals sorted by assignment to the first cluster (Fig. S4), the two Korean and the two Japanese populations have the majority of individuals belonging to the first cluster. In the four Han Chinese populations a few individuals belong to that first cluster but most individuals show some attributions to both clusters with an apparently continuous range of attribution. In the remaining East Asians, essentially Southeast Asians, most individuals show a primary assignment to the second cluster with some showing diverse partial assignments, including to the Pacific cluster.

The Pacific populations form a distinct cluster in the STRUCTURE, PCA, and LS tree analyses. The two population samples from Papua New Guinea are the most distinct, consistent with them being the only Melanesian samples in the study. The remainder of the Pacific populations show partial similarity to the East Asians.

The current results illustrate the information provided by autosomal markers. MHs provide an overall level of genetic relationship that the sex-specific markers (Y DNA and mt DNA) do not. Each of the sex-specific types of markers provides a single gene tree of the Y or mtDNA for each individual independently whereas each MH provides a separate and independent view of history and relationships of populations. MHs differ from the other category of autosomal loci with multiple alleles, short tandem repeat (STR) loci, in that MHs have much greater mutational stability with mutation rates on the order of 10^−8^ compared to STRs that have mutation rates on the order of 10^−3^. Extant variation is largely the result of pre-existing mutation and recombination events with random genetic drift as the primary null hypothesis accounting for allele frequency variation among populations. The sex-specific markers (Y and mtDNA), provide separate patterns of genetic relationships that can differ from the mult-locus estimates of the autosomal MHs.

Showing relationships among a sample of individuals in a population requires large numbers of SNPs for accuracy^54^. Logic argues that fewer microhaplotypes will be required because of their multiallelic nature^55^. The tradeoff in numbers will require more study. This large dataset with multiple MH loci supports the global distinctiveness of the North Asia populations in a way that has not been shown previously. Similarly, some of the MHs and analyses show variation among the several North Asia populations in ways that highlight the use of different methods with different underlying assumptions. We have used three of the most commonly used methods of analyzing population relationships based on autosomal markers: STRUCTURE, PCA, and Tree analysis. Other approaches to data analysis usually involve both different underlying genetic models^44^, such as specific patterns of admixture^56^, and more computer intensive methods; they are beyond the scope of this study. However, with all the data publically available it is possible for others to consider such analyses.

Our analyses confirm with microhaplotypes the general finding of 6 large “continental” biogeographic clusters of human populations. The analyses based on this set of 58 multi-allelic polymorphisms demonstrate the potential value of microhaplotypes to explore genetic relationships of populations within these “continental” clusters. These particular loci demonstrate distinct clusters of populations within Northern Europe, within North Asia, and within East Asia. More refined population relationships will certainly emerge as more and better microhaplotypes are identified that can help differentiate populations within and between the diverse geographical zones inhabited by human populations and illuminate their historical relationships. Panels of microhaplotypes have much to offer in a variety of genetic, anthropological, forensic, and medical applications.

## Electronic Resources Cited

ALFRED https://alfred.med.yale.edu. 

## Supplementary Information

Supplementary Information.

## Data Availability

The microhaplotype genotype data supporting the findings of this study can be accessed in the Zenodo archive at https://doi.org/10.5281/zenodo.4681100 for three North Asian population samples and at https://doi.org/10.5281/zenodo.4658892 for the 72 other Kidd lab population samples. All genotype profiles are anonymous.

## Abbreviations

1 KG: Thousand Genomes Consortium
A_e_: Effective number of alleles
AG: GenomeAsia 100 K Consortium
I_n_: Informativeness
LS: Least squares
MCMC: Markov Chain Monte Carlo
MH: Microhaplotypes
NJ: Neighbor joining
PCA: Principal component analysis
