# Supplementary material for: North Asian population relationships in a global context
Source: Sci Rep. 2022 May 4;12:7214. doi: 10.1038/s41598-022-10706-x (PMC9068624; doi:10.1038/s41598-022-10706-x)

Supplementary Information

for manuscript submitted to Scientific Reports

North Asian population relationships in a global context

Kenneth K. Kidd[1*], Baigalmaa Evsanaa[2], Ariunaa Togtokh[2], Jane E. Brissenden[3], Janet M. Roscoe[4,5], Mustafa Dogan[6], Pavlos I. Neophytou[7], Cemal Gurkan[8,9], Ozlem Bulbul[10], Lotfi Cherni[11,12], William C. Speed[1], Michael Murtha[1], Judith R. Kidd[1], Andrew J. Pakstis[1]

[1] Department of Genetics, Yale University School of Medicine, New Haven, CT 06520

[2] Department of Nephrology, Mongolian National University of Medical Sciences, Ulaanbaatar, Mongolia

[3] Independent Scientist, Toronto, Ontario, Canada

[4] Department of Medicine, University of Toronto, Toronto, Ontario, Canada

[5] The Scarborough Hospital, Toronto, Ontario, Canada

[6] Department of Genetics and Bioengineering, International Burch University, Sarajevo, Bosnia and Herzegovina

[7] Mendel Center for Biomedical Sciences, Egkomi, Nicosia, Cyprus

[8] Turkish Cypriot DNA Laboratory, Committee on Missing Persons in Cyprus Turkish Cypriot Member Office, Nicosia, North Cyprus, Turkey

[9] Dr. Fazıl Küçük Faculty of Medicine, Eastern Mediterranean University, Famagusta, North Cyprus, Turkey

[10] Institute of Forensic Science, Istanbul University--Cerrahpasa, Istanbul, 34500 Turkey

[11] Laboratory of Genetics, Immunology and Human Pathologies, Faculty of Sciences of Tunis, University of Tunis El Manar, 2092 Tunis, Tunisia

[12] Higher Institute of Biotechnology of Monastir, Monastir University, 5000 Monastir, Tunisia

Index of Supplementary Table and Figure Legends

Table S1. The definitions of the 58 microhaplotypes.

Figure S1. The distribution of A_e values for 58 microhaps evaluated on 122 populations.

Figure S2. STRUCTURE results for 122 populations at K=7 to 9. a) By individuals. b) By population averages.

Figure S3. STRUCTURE results overview of individual bar plots for highest likelihood runs from K=2 to 11 for the 74 population analyses.

Figure S4. STRUCTURE results by individual for 74 populations at K=7 through K=9.

Figure S5. The likelihood values for the twenty runs at each K for the 122 population STRUCTURE analyses for K=2 through K=12.

Figure S6. The likelihood values for the twenty 74-population STRUCTURE runs at each K for K=2 through K=11.

Figure S7. STRUCTURE results by individual for 15 populations at K=5 through K=7.

Figure S8. The likelihood values for the twenty 15-population STRUCTURE runs at each K for K=2 through K=11.

Table S1. The definitions of the 58 microhaplotypes.

Chr	Number of SNPs	Microhaplotype Name	Extent (base pairs)	rs-numbers of SNPs included	Nucleotide positions (Build 37)
1	4	mh01KK-001	260	rs4648344<br>rs6663840<br>rs58111155<br>rs6688969	3743132<br>3743319<br>3743350<br>3743391
1	2	mh01KK-002	18	rs4528199<br>rs6604596	216634428<br>216634445
1	4	mh01KK-106	171	rs12123330<br>rs16840876<br>rs56212601<br>rs4468133	4227464<br>4227561<br>4227624<br>4227634
1	4	mh01KK-117	187	rs17413714<br>rs2772234<br>rs1610401<br>rs1610400	204633340<br>204633397<br>204633500<br>204633526
1	2	mh01KK-135	289	rs61822416<br>rs12036383	234206033<br>234206321
1	3	mh01KK-172	226	rs3128342<br>rs3766176<br>rs1887284	1486834<br>1486903<br>1487059
1	4	mh01KK-205	155	rs11810587<br>rs1336130<br>rs1533623<br>rs1533622	18722692<br>18722713<br>18722801<br>18722846
1	3	mh01KK-211	149	rs2490423<br>rs16835127<br>rs2341465	161955656<br>161955758<br>161955804
2	3	mh02KK-003	125	rs260694<br>rs11123719<br>rs11691107	109586313<br>109586371<br>109586437
2	2	mh02KK-105	22	rs2280355<br>rs2280356	97366303<br>97366324
2	4	mh02KK-134	104	rs12469721<br>rs3101043<br>rs3111398<br>rs72623112	161079411<br>161079435<br>161079450<br>161079514
2	2	mh02KK-202	140	rs13422174<br>rs12464185	107648173<br>107648312
2	2	mh02KK-215	43	rs2011946<br>rs16832624	136817616<br>136817658
3	2	mh03KK-006	64	rs1919550<br>rs9873644	121364173<br>121364236

3	2	mh03KK-020	220	rs4683510<br>rs12494698	140285115<br>140285334
3	4	mh03KK-150	185	rs1225051<br>rs1225050<br>rs1225049<br>rs1225048	131645972<br>131646001<br>131646087<br>131646156
4	2	mh04KK-010	35	rs3135123<br>rs495367	1986938<br>1986972
4	5	mh04KK-013	201	rs13131164<br>rs3775866<br>rs11725922<br>rs3775867<br>rs17088476	68444102<br>68444180<br>68444192<br>68444257<br>68444302
4	3	mh04KK-017	153	rs4699748<br>rs2584461<br>rs1442492	100321443<br>100321573<br>100321595
4	4	mh04KK-030	115	rs16844737<br>rs4916615<br>rs1884412<br>rs1884411	3666370<br>3666403<br>3666471<br>3666484
5	2	mh05KK-062	19	rs870348<br>rs870347	6845017<br>6845035
5	3	mh05KK-120	276	rs2278007<br>rs16891982<br>rs35398	33951551<br>33951693<br>33951826
6	3	mh06KK-030	100	rs10949381<br>rs675934<br>rs607341	16801767<br>16801783<br>16801866
6	2	mh06KK-031	159	rs10455681<br>rs10455682	69802502<br>69802660
6	2	mh06KK-101	187	rs9356632<br>rs2180052	170589803<br>170589989
9	3	mh09KK-033	78	rs10815466<br>rs9408671<br>rs17431629	680714<br>680763<br>680791
9	2	mh09KK-035	194	rs3118582<br>rs10776839	137417115<br>137417308
9	4	mh09KK-152	142	rs10867949<br>rs4282648<br>rs10780576<br>rs7046769	85808649<br>85808708<br>85808730<br>85808790
9	3	mh09KK-153	113	rs10125791<br>rs2987741<br>rs7047561	103969740<br>103969775<br>103969852
9	2	mh09KK-161	104	rs4741823<br>rs16932430	344229<br>344332

10	5	mh10KK-163	260	rs3814588<br>rs6602026<br>rs3814589<br>rs3814590<br>rs9423466	3162410<br>3162423<br>3162486<br>3162525<br>3162669
10	5	mh10KK-169	292	rs10906617<br>rs10796164<br>rs10796165<br>rs17154765<br>rs10796166	14208361<br>14208510<br>14208588<br>14208611<br>14208652
10	3	mh10KK-170	190	rs2250841<br>rs2250840<br>rs12359688	78910042<br>78910074<br>78910231
11	3	mh11KK-092	296	rs7928046<br>rs1943620<br>rs1807939	113065024<br>113065293<br>113065319
11	3	mh11KK-103 †	146	rs1107162<br>rs2075654<br>rs1079727	113289037<br>113289066<br>113289182
11	4	mh11KK-180	194	rs12802112<br>rs28631755<br>rs7112918<br>rs4752777	1690791<br>1690911<br>1690969<br>1690984
12	4	mh12KK-202	154	rs10506052<br>rs4931233<br>rs10506053<br>rs4931234	30170229<br>30170306<br>30170359<br>30170382
13	2	mh13KK-047	166	rs806301<br>rs2066700	50887560<br>50887725
13	4	mh13KK-217	193	rs7320507<br>rs9562648<br>rs9562649<br>rs2765614	46865930<br>46865970<br>46866084<br>46866122
13	3	mh13KK-225	97	rs4884651<br>rs9529023<br>rs7329287	66712732<br>66712790<br>66712828
13	2	mh13KK-226	118	rs721367<br>rs2892698	95546650<br>95546767
14	2	mh14KK-101	96	rs28529526<br>rs10134526	106009477<br>106009572
15	2	mh15KK-066	75	rs1063902<br>rs4219	52484950<br>52485024
15	2	mh15KK-067	122	rs701463<br>rs701464	46870734<br>46870855
15	2	mh15KK-095	100	rs2433354<br>rs2459391	48414969<br>48415068

16	4	mh16KK-049 †	174	rs9937467<br>rs17670098<br>rs12929083<br>rs9926495	7209208<br>7209247<br>7209311<br>7209381
16	2	mh16KK-053	12	rs201075024<br>rs11150606	31099000<br>31099011
16	2	mh16KK-061	50	rs4559917<br>rs6540049	87481379<br>87481428
16	2	mh16KK-096	38	rs1805007<br>rs885479	89986117<br>89986154
16	4	mh16KK-302	114	rs1395579<br>rs1395580<br>rs1395582<br>rs9939248	7587734<br>7587746<br>7587804<br>7587847
17	2	mh17KK-052	187	rs1059504<br>rs8327	43472321<br>43472507
17	3	mh17KK-105	130	rs1052553<br>rs17652121<br>rs11568305	44073889<br>44073973<br>44074018
17	4	mh17KK-272	131	rs2934897<br>rs7207239<br>rs16955257<br>rs7212184	52942428<br>52942456<br>52942491<br>52942558
20	2	mh20KK-035	31	rs6136957<br>rs13044611	2069345<br>2069375
20	3	mh20KK-058	106	rs6122890<br>rs6095836<br>rs6012881	48844260<br>48844293<br>48844365
21	4	mh21KK-316	135	rs961302<br>rs17002090<br>rs961301<br>rs2830208	27782968<br>27782992<br>27783039<br>27783102
21	4	mh21KK-324	159	rs6518223<br>rs2838868<br>rs7279250<br>rs8133697	46714549<br>46714641<br>46714692<br>46714707
22	2	mh22KK-069	79	rs8137373<br>rs2235845	41729216<br>41729294

Note:
† indicates two microhaps—mh11KK-103 and mh16KK-049--that each have one less defining SNP in analyses reported here compared to defining SNPs listed in ALFRED.

Figure S1. The distribution of A_e values for 58 microhaps evaluated on 122 populations.

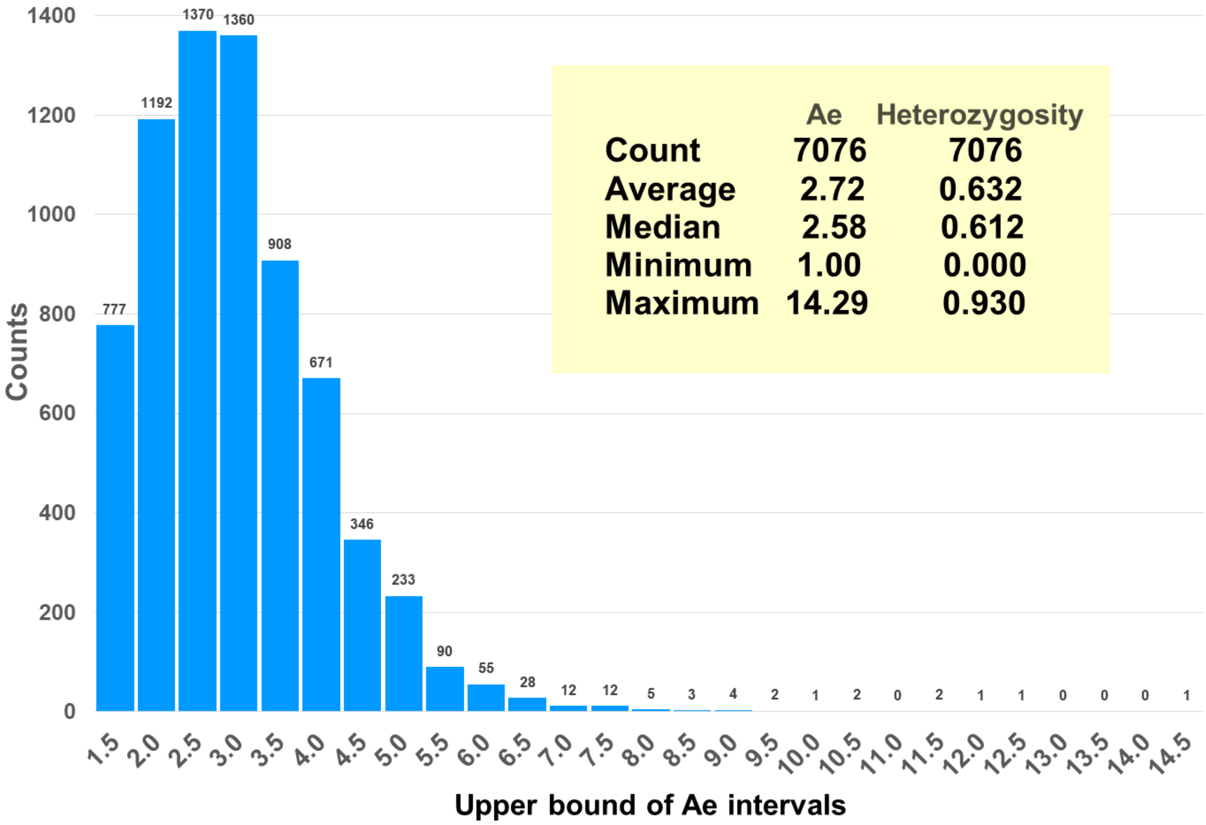

Figure S2. STRUCTURE results for 122 populations at K=7 to 9. a) By individuals. b) By population averages.

Figure S2a.

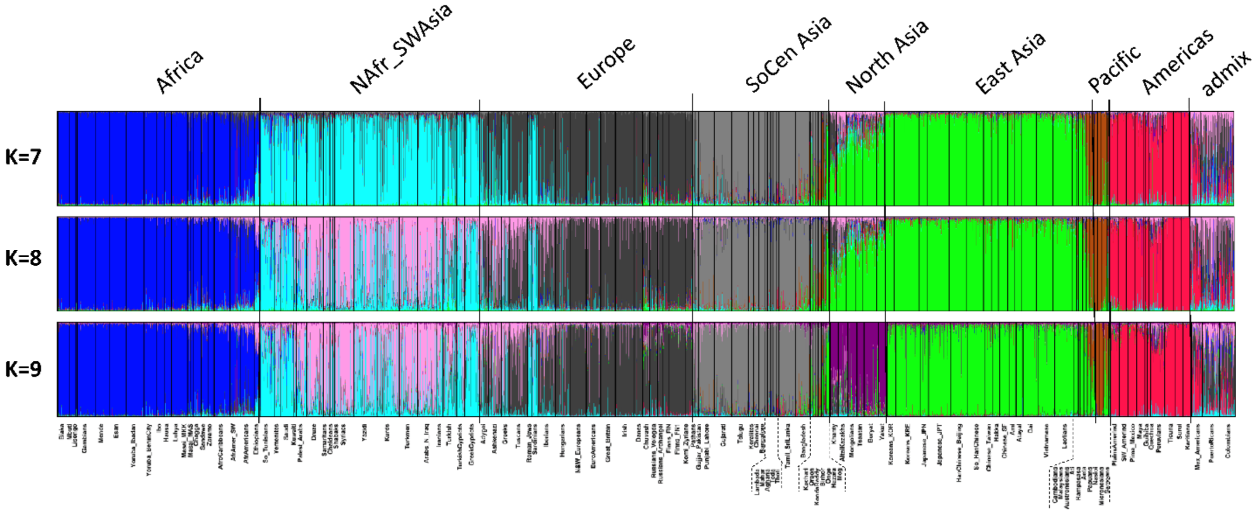

Figure S2b.

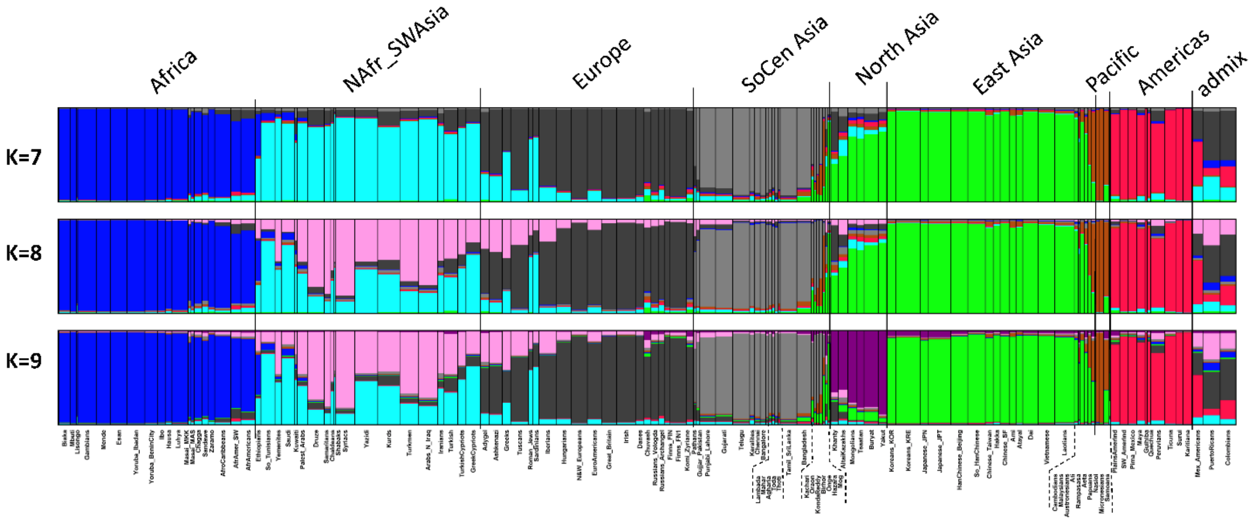

Figure S3. STRUCTURE results overview of individual bar plots for highest likelihood runs from K=2 to 11 for the 74 population analyses.

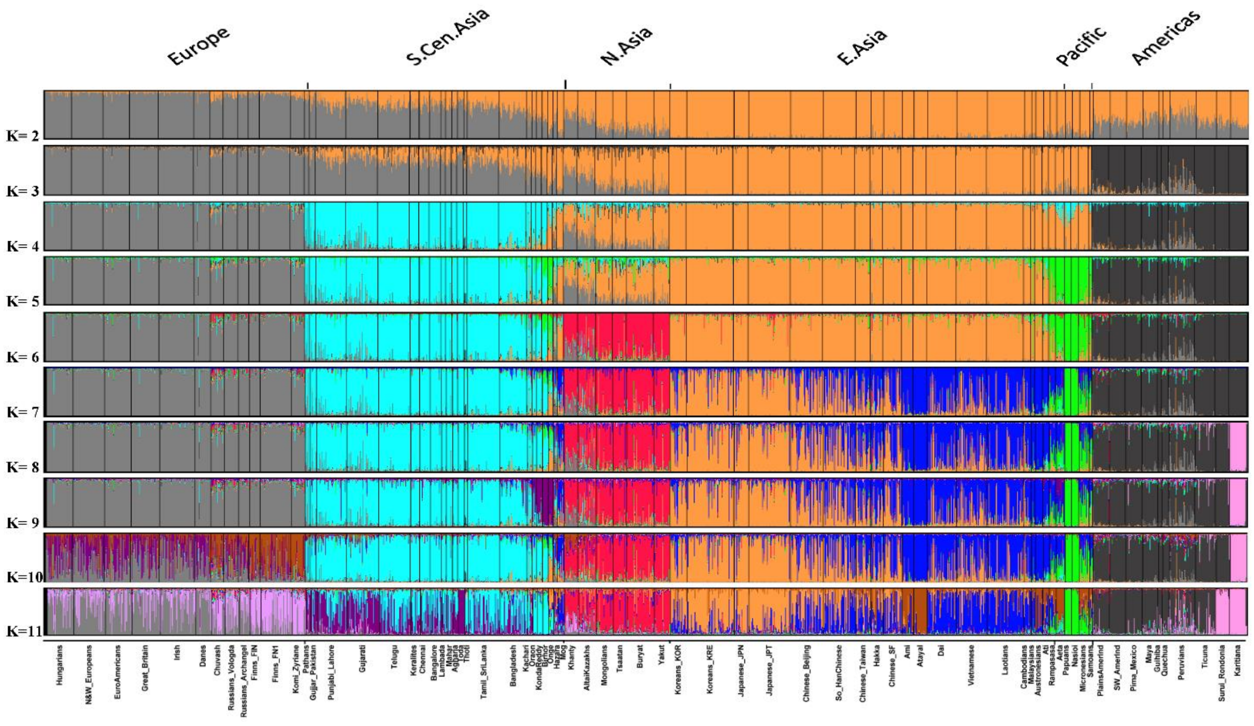

Figure S4. STRUCTURE results by individual for 74 populations at K=7 through K=9.

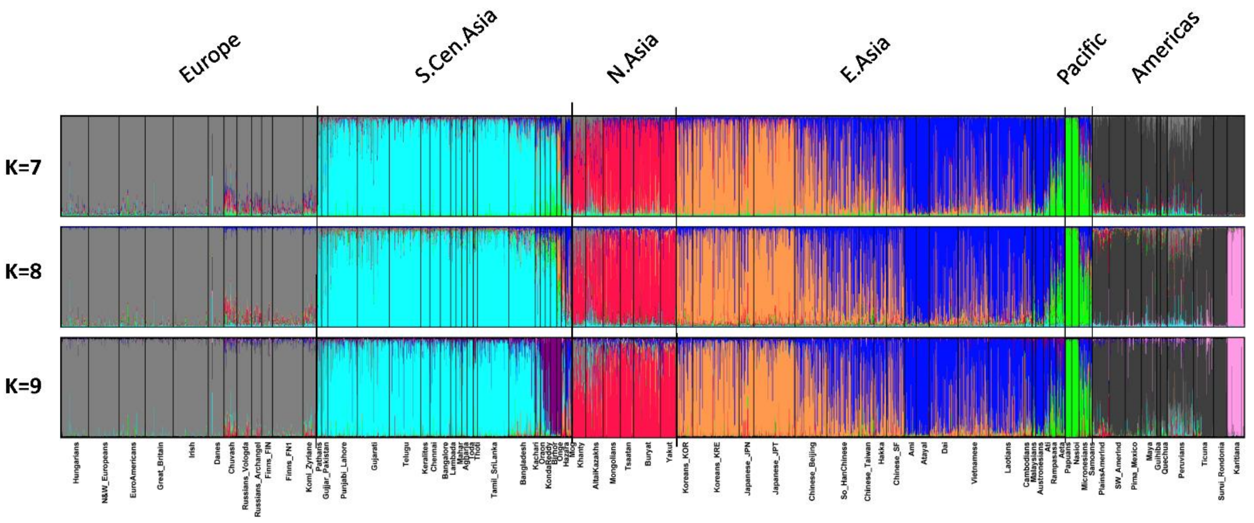

Figure S5. The likelihood values for the twenty runs at each K for the 122 population STRUCTURE analyses for K=2 through K=12.

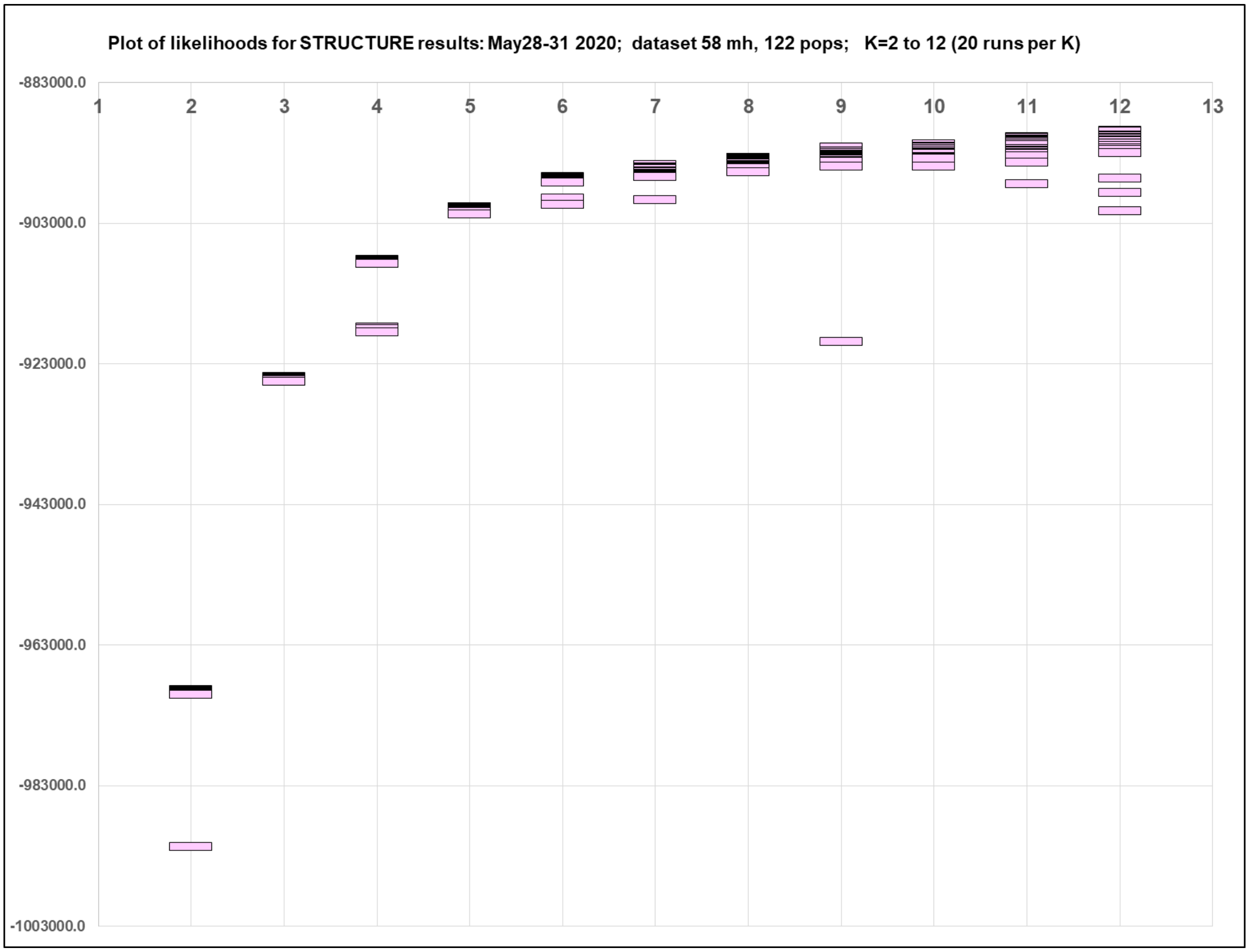

Figure S6. The likelihood values for the twenty 74-population STRUCTURE runs at each K for K=2 through K=11.

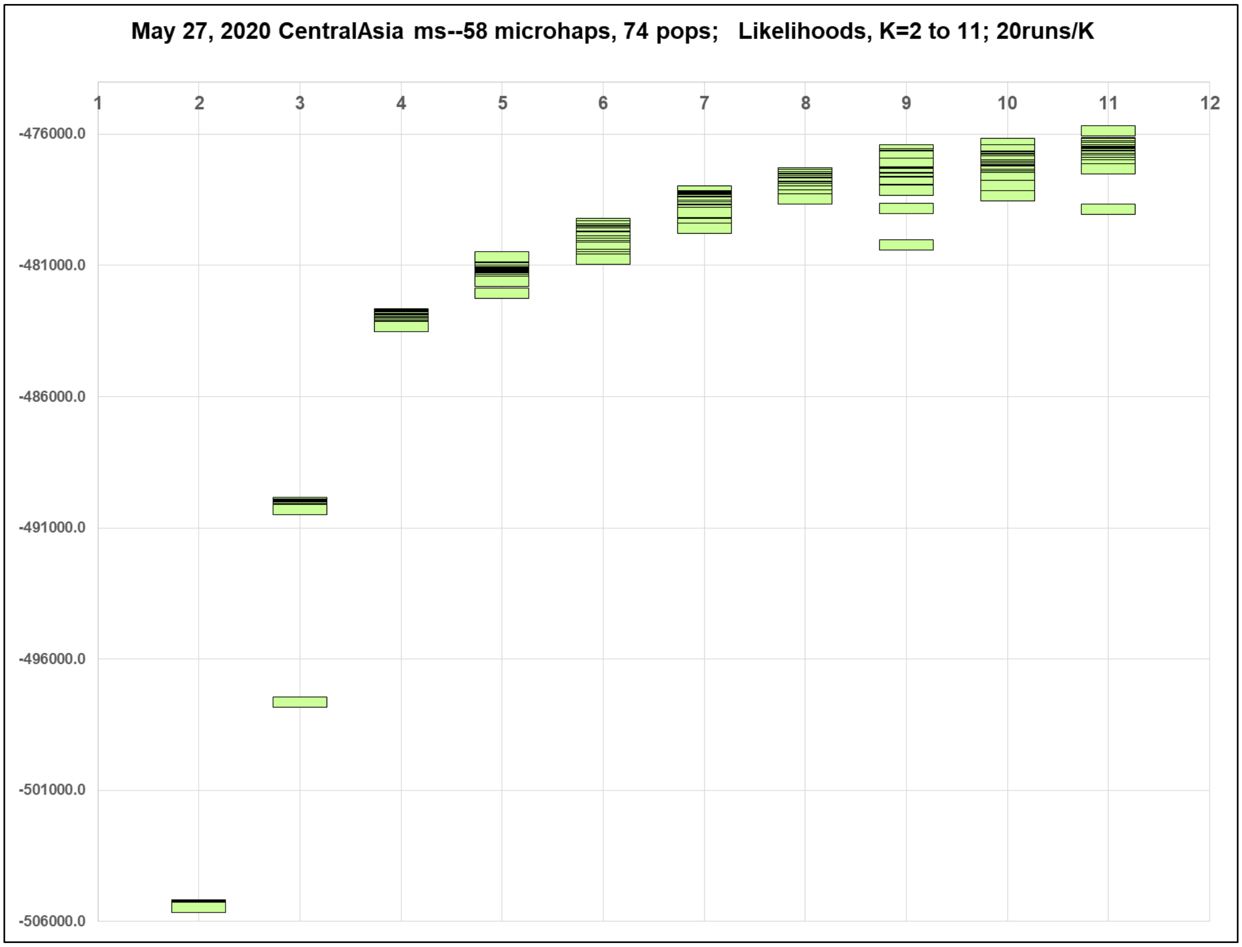

Figure S7. STRUCTURE results by individual for 15 populations at K=5 through K=7.

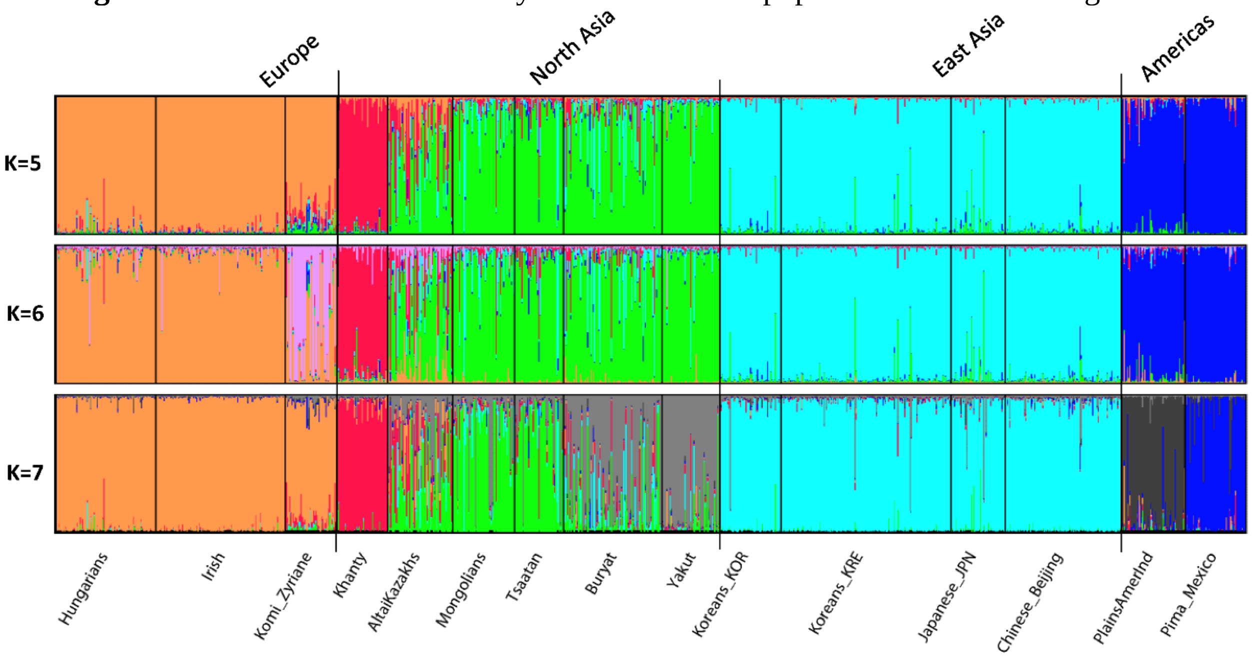

Figure S8. The likelihod values for the twenty 15-population STRUCTURE runs at each K for K=2 through K=11.

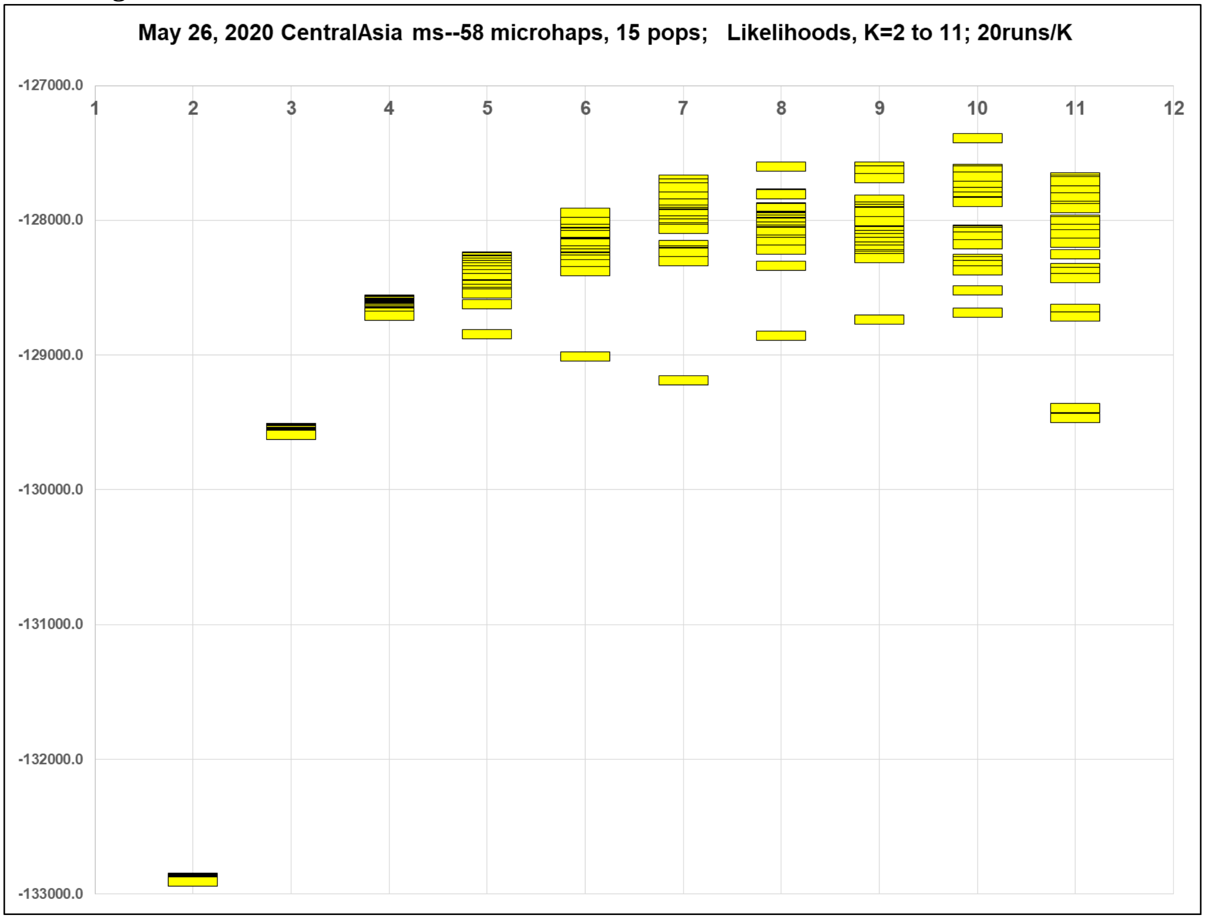

Supplement: Supplementary file 1 — Supplementary Information. [file 41598_2022_10706_MOESM1_ESM.pdf]
